# A cognitive neural circuit biotype of depression showing functional and behavioral improvement after transcranial magnetic stimulation in the B-SMART-fMRI trial

**DOI:** 10.1038/s44220-024-00271-9

**Published:** 2024-07-05

**Authors:** Leonardo Tozzi, Claire Bertrand, Laura Michele Hack, Timothy Lyons, Alisa Marie Olmsted, Divya Rajasekharan, TeChieh Chen, Yosef A. Berlow, Jerome A. Yesavage, Kelvin Lim, Michelle R. Madore, Noah S. Philip, Paul Holtzheimer, Leanne Maree Williams

**Affiliations:** 1 Department of Psychiatry and Behavioral Sciences, Stanford University School of Medicine, Stanford, CA, USA.; 2 Mental Illness Research, Education and Clinical Center, VA Palo Alto Health Care System, Palo Alto, CA, USA.; 3 National Center for PTSD, VA Medical Center, US Department of Veterans Affairs, White River Junction, VT, USA.; 4 Department of Psychiatry and Human Behavior, Alpert Medical School of Brown University, Providence, RI, USA.; 5 VA RR&D Center for Neurorestoration and Neurotechnology, VA Providence Healthcare System, Providence, RI, USA.; 6 Department of Psychiatry and Behavioral Sciences, University of Minnesota Medical School, Minneapolis, MN, USA.; 7 Minneapolis VA Health Care System, Minneapolis, MN, USA.; 8 Geisel School of Medicine at Dartmouth, Hanover, NH, USA.; 9 These authors contributed equally: Claire Bertrand, Laura Michele Hack, Timothy Lyons, Alisa Marie Olmsted, Divya Rajasekharan.; 10 These authors jointly supervised this work: Kelvin Lim, Michelle Madore, Noah S. Philip, Paul Holtzheimer, Leanne Maree Williams.

## Abstract

We previously identified a cognitive biotype of depression characterized by treatment resistance, impaired cognitive control behavioral performance and dysfunction in the cognitive control circuit, comprising the dorsolateral prefrontal cortex (dLPFC) and dorsal anterior cingulate cortex (dACC). Therapeutic transcranial magnetic stimulation (TMS) to the left dLPFC is a promising option for individuals whose depression does not respond to pharmacotherapy. Here, 43 veterans with treatment-resistant depression were assessed before TMS, after early TMS and post-TMS using functional magnetic resonance imaging during a Go–NoGo paradigm, behavioral cognitive control tests and symptom questionnaires. Stratifying veterans at baseline based on task-evoked dLPFC–dACC connectivity, we demonstrate that TMS-related improvement in cognitive control circuit connectivity and behavioral performance is specific to individuals with reduced connectivity at baseline (cognitive biotype +), whereas individuals with intact connectivity at baseline (cognitive biotype −) did not demonstrate significant changes. Our findings show that dLPFC–dACC connectivity during cognitive control is both a promising diagnostic biomarker for a cognitive biotype of depression and a response biomarker for cognitive improvement after TMS applied to the dLPFC.

Major depressive disorder (MDD) is highly prevalent and the leading cause of disability^1^. Despite the availability of multiple treatment options, we lack tests for identifying which treatment is most likely to benefit each individual. Most patients cycle through multiple trial- and-error treatment trials, often over years and some patients become resistant to treatment. The need for precision treatments is especially urgent for veterans given the high prevalence of depression with comorbid conditions and a higher rate of suicide compared with the civilian population^2^. This need was corroborated by the launch of the Scott Hannon Initiative for Precision Mental Health, which directs the US Department of Veterans Affairs to utilize methods such as functional neuroimaging to tailor treatments to individuals^3^.

A growing pool of studies highlight that cognitive impairments occur with depression and are a major contributor to chronic disability across the lifespan of affected individuals^4–7^. These cognitive impairments can persist despite overall symptom relief during early-to-mid adulthood^8^ and similarly predict poor response to antidepressant medication in later-life depression^9,10^. We previously identified a cognitive biotype of depression in 27% of individuals with depression^11^. At the pretreatment baseline this biotype is characterized by impaired performance on tests of cognitive control, greater disability and dysfunction of the brain’s cognitive control circuit, in particular in the dorsolateral prefrontal cortex (dLPFC) and dorsal anterior cingulate cortex (dACC)^11^. Following treatment with pharmacotherapy, this biotype also shows comparatively low response and remission rates^11^. Furthermore, we found that pretreatment task-related connectivity of the cognitive control circuit also predicts pharmacotherapy outcomes and changes proportionally with treatment^12^. Thus, pretreatment cognitive control circuit function offers promise as a biomarker for stratification in precision mental health to help select those individuals who are less likely to respond to pharmacotherapy alone and may benefit from different therapeutic approaches.

Therapeutic transcranial magnetic stimulation (TMS) has emerged as a promising therapeutic option for individuals whose depression does not respond to pharmacotherapy alone and who are at risk of chronic disability^13^. It involves the non-invasive application of electromagnetic pulses to specific regions of the brain, aimed at rectifying the neural circuitry dysfunction underlying pharmacoresistant depression^14–16^. In the treatment of MDD, TMS is most frequently used to induce an electric field that results in the depolarization of neurons in the dLPFC^17,18^. The most common protocol for TMS delivery is once-daily treatments for up to 30 sessions, followed by a taper^13^. However, a number of novel or alternative approaches have evolved in recent years, including the use of different parameters or protocols (for example, theta burst stimulation or accelerated TMS; reviewed elsewhere^19–22^).

Having shown that functional connectivity within the cognitive control circuit is a promising candidate biomarker for treatment response in MDD and that TMS targets the dLPFC, a core region within this circuit, we hypothesized that functional connectivity of the dLPFC would provide a promising biomarker to prospectively identify patients who are non-responsive to pharmacotherapy and likely to benefit from clinical TMS. In the present study we used functional magnetic resonance imaging (fMRI) embedded within a large multisite clinical TMS program. We used a pre-specified stratified precision medicine design to quantify functional connectivity before the commencement of TMS over the dLPFC^23^. Informed by previous work that indicates that a large portion of symptom reduction may occur early^24^, we performed fMRI early in the TMS treatment process and again at the end of treatment to determine the timing and extent of change in brain connectivity dependent on the extent of pretreatment impairment. We evaluated whether early changes in cognitive control connectivity determine subsequent changes in both cognitive behavior and clinical symptoms, and whether the extent of the change in connectivity correlates with the magnitude of the clinical response.

A focus of the present study was the quantification of cognitive control circuit connectivity engaged by cognitive task demands. We focused specifically on connectivity between the dLPFC and dACC, another core region of the cognitive control circuit^25,26^, which is also impaired in the cognitive control biotype^11^. Previous neuroimaging studies investigating how connectivity between the dLPFC and dACC relates to TMS treatment response have largely done so using resting-state fMRI. These studies suggest that TMS of the dLPFC might improve symptoms of depression by affecting the functional coupling between dLPFC and regions of the anterior cingulate cortex (ACC)^15^, although the putative therapeutic mechanism of TMS remains under study.

Consistent with the pre-registered aims of the present TMS study^23^, we first tested the primary hypothesis that task-related dLPFC–dACC connectivity within the cognitive control circuit would change in a session-dependent manner—from the pre-TMS baseline to post early TMS treatment through to the end of treatment (primary outcome)—and that the extent of this change would be related to baseline stratification by connectivity. We then tested the hypothesis that the magnitude of change in cognitive control connectivity would be associated with corresponding change in cognitive performance as well as in overall symptom severity (secondary outcomes).

Our findings demonstrate that before commencing TMS we can stratify individuals into those who have poor dLPFC–dACC connectivity (cognitive biotype +) and those with relatively intact connectivity (cognitive biotype −). Importantly, this stratification is clinically meaningful for determining the subsequent trajectory of the response to TMS. Specifically, individuals with impaired cognitive control circuit function before commencing TMS show TMS-related improvement in connectivity and this improvement largely occurs after the early sessions of TMS. Consistent with our hypotheses, early connectivity improvement corresponds with improved cognitive performance and this association is dependent on pretreatment baseline stratification. Furthermore, connectivity changes statistically mediate improved performance. By elucidating how specific neural mechanisms underlying cognitive impairments in depression are modulated by TMS and for whom, our study is an important advance towards the development of personalized treatment strategies, with a particular emphasis on prospective identification of who might benefit from TMS for improving cognitive function.

## Results

### Stratification according to cognitive control circuit connectivity

Baseline stratification was based on left dLPFC–dACC connectivity within the cognitive control circuit before TMS treatment (Table 1). The cognitive biotype (*n* = 26) was defined as participants who had hypoconnectivity of the left dLPFC and the dACC during the Go–NoGo task relative to a healthy norm. We labeled these participants as ‘cognitive biotype +’. By contrast, those not in the cognitive biotype (*n* = 17) were defined as participants who have relatively intact connectivity of the dLPFC–dACC. We labeled these participants as ‘cognitive biotype −’. The connectivity cut-off values for cognitive biotypes + and − were, respectively, <0 and ≥0, after expressing the connectivity values relative to a healthy norm (more details in Methods).

**Fig. 1 |:**
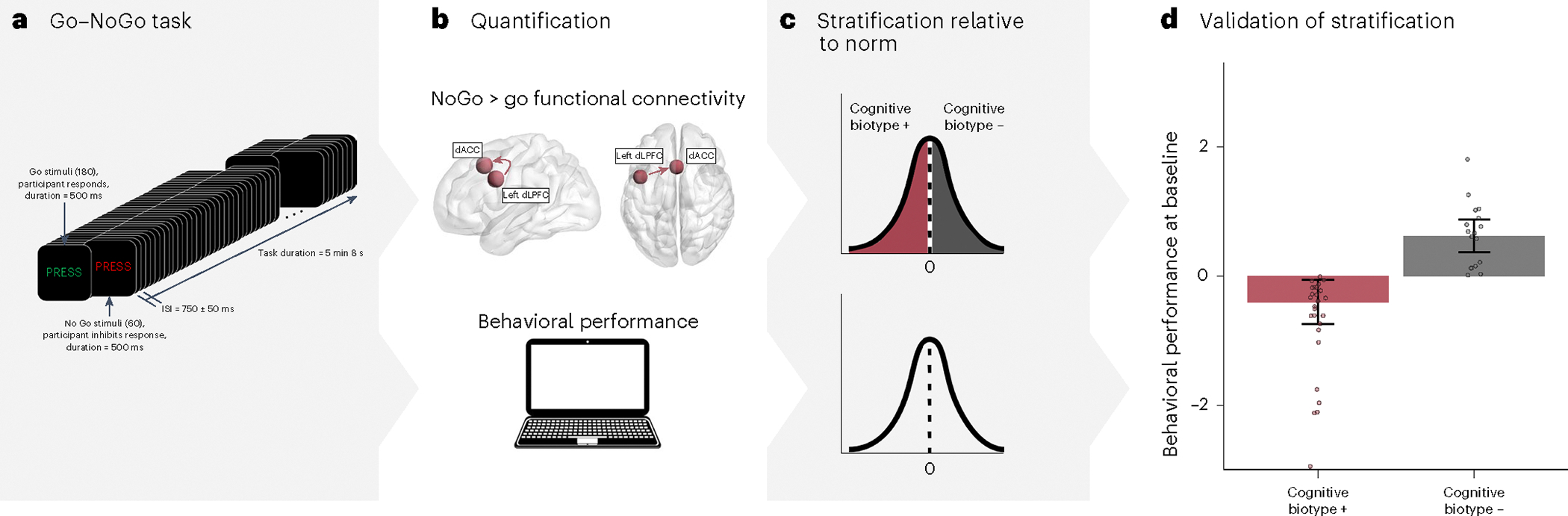
Stratification and validation of the cognitive biotype groups defined by functional connectivity within the cognitive control circuit. **a**, Task fMRI data were collected during a Go–NoGo task that requires response inhibition and is designed to engage the cognitive control circuit. ISI, interstimulus interval. **b**, From the scan we quantified connectivity to define regions of the cognitive control circuit, the left dLPFC and dACC, for the contrast of NoGo>Go (top). Left dLPFC–dACC connectivity was quantified for individual participants and connectivity scores were expressed in s.d. units relative to a healthy reference dataset. Cognitive behavioral performance was assessed using the same Go–NoGo task conducted outside of the scanner (bottom). Cognitive performance was quantified as the composite of commission and omission errors, and also expressed in s.d. units relative to a healthy reference dataset. **c**, We then stratified the sample into two biotypes based on their pretreatment baseline left dLPFC–dACC connectivity. The cognitive biotype (*n* = 26) was defined as participants with hypoconnectivity (<0) relative to a healthy norm, which we termed cognitive biotype + and those not in the cognitive biotype (*n* = 17) were defined as participants with intact connectivity (≥0), which we termed cognitive biotype − (top). **d**, Stratification on the basis of baseline cognitive control circuit connectivity was validated by biotype differences in baseline cognitive performance. Veterans in the cognitive biotype + group (*n* = 26) were distinguished from cognitive biotype − (*n* = 17) by significantly poorer accuracy for the composite measure of commission and omission errors (cognitive biotype + versus cognitive biotype −; *t* = −2.127, *P* = 0.041, 95% CI = [−2.003; −0.042]). The mean is shown as bars, the s.e.m. are shown as whiskers and individual data points are shown as dots.

Baseline stratification by cognitive control circuit function was reflected in a corresponding stratification according to behavioral performance in computerized tests of cognitive control performance collected using the cognitive test battery WebNeuro. At baseline, veterans in the cognitive biotype + group were distinguished from the cognitive biotype − veterans by significantly worse cognitive control performance on the Go–NoGo test (*t*-statistic (*t*) = −2.127, degrees of freedom (d.f.) =31.398, *P* = 0.041, Cohen’s *d* = −0.759, 95% confidence interval (CI) = [−2.003; −0.042]; Fig. 1).

### TMS restores cognitive control circuit connectivity

**Fig. 2 |:**
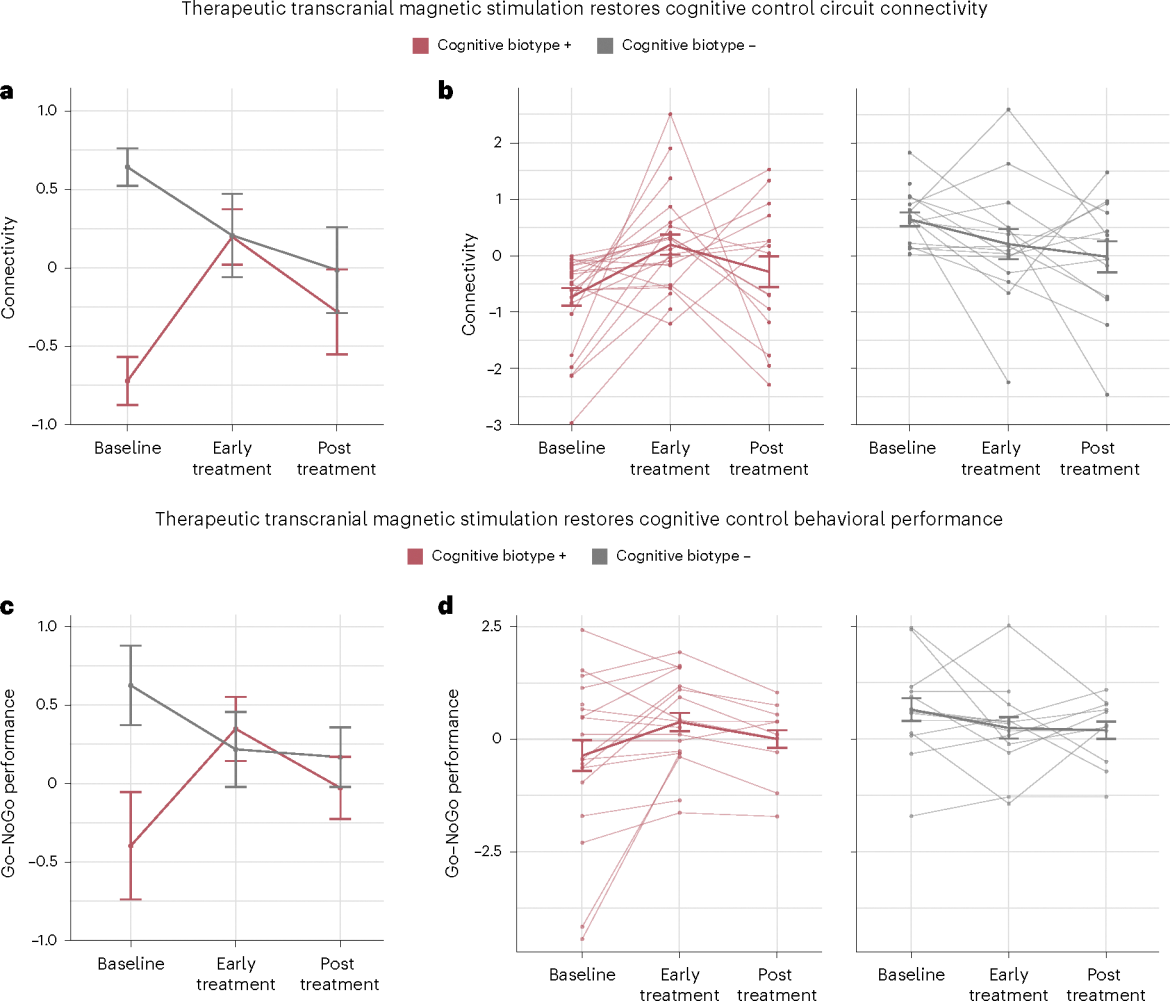
TMS restores impaired cognitive control circuit connectivity and cognitive control behavioral performance in a session-dependent manner. **a**–**d**, Changes in cognitive control circuit connectivity (**a,b**) and behavioral performance (**c,d**) were analyzed using a linear mixed-effects model to test for the effects of biotype, session and their interaction while adjusting for time (in days) from baseline on the scan date and repeated measure effects. Data are mean and s.e.m. **a**, TMS produced an early improvement in cognitive control circuit connectivity, which was dependent on the pre-TMS stratification (biotype by session interaction, *F* = 7.485, d.f. = 2, *P* = 0.001). This early improvement was specific to the veterans in the cognitive biotype and was present both early after TMS and at the end of treatment (early versus baseline, *t* = 4.784, *P* < 0.001, *d* = 1.114, 95% CI = [0.773; 1.876]; post treatment versus baseline, *t* = 3.347, *P* = 0.001, *d* = 0.675, 95% CI = [0.661; 2.585]). **b**, Data for individual veterans in each of the two cognitive biotypes, indicated by individual data points and connected by faint lines. **c**, Cognitive control behavioral performance of both biotypes, mirroring the findings in **a**. We observed early TMS-induced improvement in cognitive control performance that was specific to the cognitive biotype + group (biotype by session interaction, *F* = 4.178, d.f. = 2, *P* = 0.021) and this improvement was present both early after TMS and at the end of treatment (early versus baseline, *t* = 3.862, *P* < 0.001, *d* = 1.075, 95% CI = [0.513; 1.622]; post treatment versus baseline, *t* = 3.177, *P* = 0.002, *d* = 0.767, 95% CI = [0.764; 3.343]). **d**, Go–NoGo performance values for individual veterans in both biotypes, indicated as data points and connected by faint lines. All statistical tests were two-sided and not adjusted for multiple comparisons.

TMS produced an early improvement in cognitive control circuit connectivity that was dependent on pre-TMS stratification. This early improvement was specific to veterans in cognitive biotype + and persisted until the end of treatment. This effect of TMS by session and baseline stratification was reflected in a significant biotype by session interaction (*F* = 7.485, d.f. = 2, *P* = 0.001; values and change in Fig. 2 and Supplementary Fig. 1, respectively, and Supplementary Table 1).

Follow-up tests showed that there was already a significant increase in connectivity after early treatment (*t* = 4.784, d.f. = 73.7, *P* < 0.001, *d* = 1.114, 95% CI = [0.773; 1.876]) for veterans in cognitive biotype +, which remained higher than baseline post treatment (*t* = 3.347, d.f. = 99.1, *P* = 0.001, *d* = 0.675, 95% CI = [0.661; 2.585]; values and change in Fig. 2 and Supplementary Fig. 1, respectively, and Supplementary Table 1). By contrast, no significant changes were observed for the cognitive biotype − group at either time point (early treatment, *t* = −0.581, d.f. = 68.7, *P* = 0.563, *d* = −0.140, 95% CI = [−0.834; 0.458]; and post treatment, *t* = 0.995, d.f. = 97.1, *P* = 0.322, *d* = 0.202, 95% CI = [−0.499; 1.501]; values and change in Fig. 2 and Supplementary Fig. 1, respectively, and Supplementary Table 1).

### TMS restores cognitive control behavioral performance

Mirroring the findings for brain connectivity, we also observed early TMS-induced improvement in cognitive control performance that was specific to veterans in the cognitive biotype + group, and this improvement persisted until the end of treatment, which was reflected in a significant biotype by session interaction (*F* = 4.178, d.f. = 2, *P* = 0.021; values and change in Fig. 2 and Supplementary Fig. 2, respectively, and Supplementary Table 2).

Follow-up tests showed that cognitive control performance had already significantly increased for the cognitive biotype + group after early treatment (*t* = 3.862, d.f. = 51.6, *P* < 0.001, *d* = 1.075, 95% CI = [0.513; 1.62]) and remained above baseline levels post treatment (*t* = 3.177, d.f. = 68.6, *P* = 0.002, *d* = 0.767, 95% CI = [0.764; 3.34]; values and change in Fig. 2 and Supplementary Fig. 2, respectively, and Supplementary Table 2). By contrast, no significant changes were observed for the cognitive biotype − group at either time point (early treatment, *t* = −0.213, d.f. = 50.6, *P* = 0.832, *d* = 0.060, 95% CI = [−0.749; 0.60]; and post treatment, *t* = 1.916, d.f. = 66.7, *P* = 0.060, *d* = −0.469, 95% CI = [−0.051; 2.47]; values and change in Fig. 2 and Supplementary Fig. 2, respectively, and Supplementary Table 2).

### Connectivity improvement mediates behavioral improvement

**Fig. 3 |:**
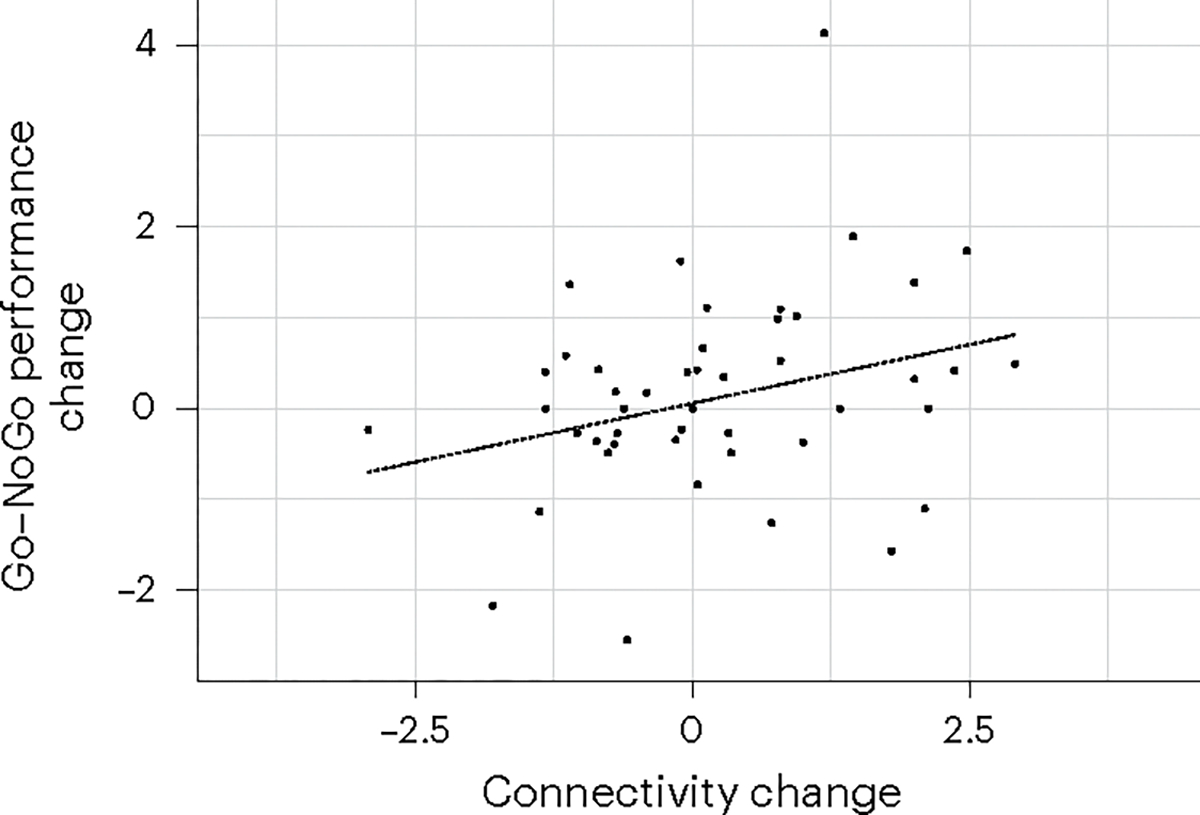
Restoration of cognitive control circuit connectivity by TMS correlates with improvement of cognitive control performance. Changes in cognitive control circuit connectivity across sessions correlated positively with changes in cognitive control performance (*r* = 0.299, two-sided *P* = 0.004).

Improvements in cognitive control circuit connectivity across sessions correlated positively with improvements in cognitive control performance (Pearson’s *r* = 0.299, *P* = 0.004, 95% CI = [0.097; 0.477]; Fig. 3).

**Fig. 4 |:**
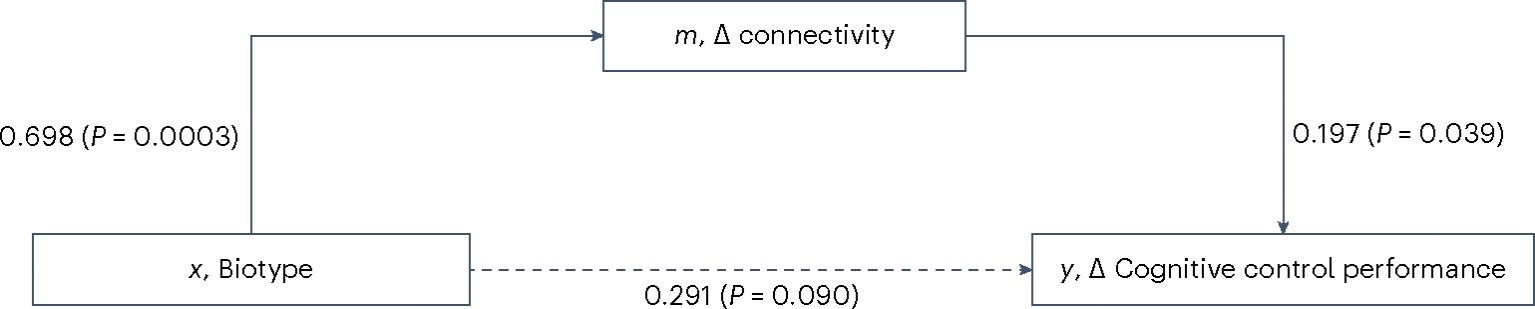
Causal mediation model linking biotype, changes from baseline in cognitive control circuit connectivity and changes from baseline in cognitive control performance following TMS. To determine whether the changes in cognitive control performance were driven by those in functional connectivity, we built a causal mediation model. In this model the independent variable was biotype (factor: cognitive biotype − and cognitive biotype +), the mediator variable was change in left dLPFC–dACC NoGo>Go connectivity from baseline and the dependent variable was change in cognitive control performance from the baseline. Average causal mediation effects and average direct effects were considered significant when *P* < 0.05. All statistical tests were two-sided and not adjusted for multiple comparisons. Δ, Change.

This association was due to the role of change in connectivity of the left dLPFC and dACC in causally mediating the change in cognitive control performance (average causal mediation effect = 0.140, 95% CI = [0.013; 0.30], *P* = 0.026; total effect = 0.431, 95% CI = [0.104; 0.740], *P* = 0.012; Fig. 4).

### TMS reduces depression symptoms regardless of connectivity

**Fig. 5 |:**
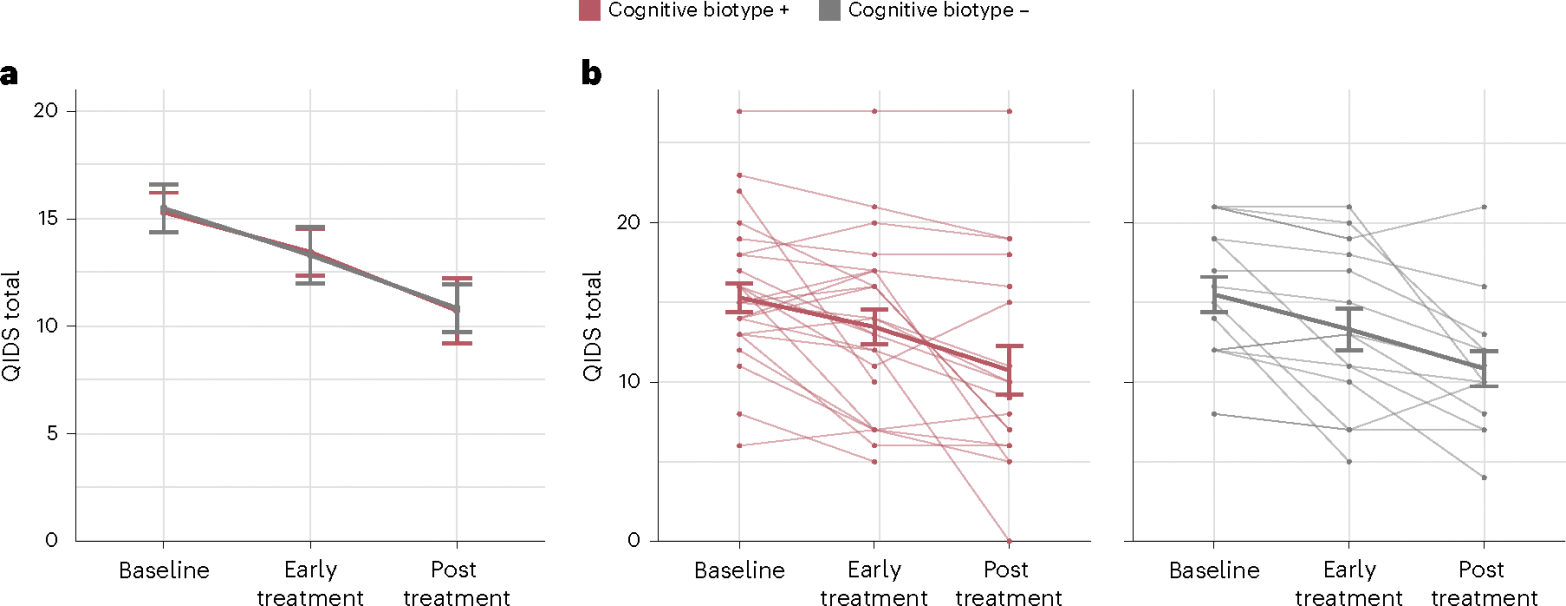
TMS reduces depression severity. **a,b**, Depression severity was measured pretreatment, post early treatment, and post treatment using the quick inventory of depression symptoms (QIDS) total score. Changes in depression severity were analyzed using a linear mixed-effects model to test for the effects of biotype, session and their interaction while adjusting for time from baseline on the scan date and repeated measure effects. The thick lines indicate the mean of depression severity and the error bars the s.e.m. **a**, TMS reduced depression severity of individuals in both cognitive biotypes (main effect of session, *F* = 4.366, d.f. = 2, *P* = 0.016). Depression severity significantly decreased from baseline to early post treatment (early versus baseline, *t* = −2.758, d.f. = 75, *P* = 0.007, 95% CI = [−3.24; −0.523]) and remained lower post treatment (post treatment versus baseline *t* = −2.337, d.f. = 103, *P* = 0.021, 95% CI = [−6.230; −0.510]). **b**, Data for individual veterans in each of the two cognitive biotypes, indicated by individual data points and connected by faint lines. All statistical tests were two-sided and not adjusted for multiple comparisons.

Depression severity was measured at three time points: pretreatment, post early treatment and post treatment. Regardless of the cognitive control connectivity at baseline, TMS resulted in reduced depression severity for all veterans (main effect of session, *F* = 4.366, d.f. = 2, *P* = 0.016; values and change in Fig. 5 and Supplementary Fig. 3, respectively, and Supplementary Table 3). A significant decrease in depression severity was already observed after early treatment (*t* = −2.758, d.f. = 75, *P* = 0.007, *d* = −0.637, 95% CI = [−3.24; −0.523]) and depression severity remained lower than baseline post treatment (*t* = −2.337, d.f. = 103, *P* = 0.021, *d* = −0.460, 95% CI = [−6.23; −0.510]; values and change in Fig. 5 and Supplementary Fig. 3, respectively, and Supplementary Table 3).

The proportion of responders post treatment did not differ between veterans in the two cognitive biotype groups (*n* = 6 cognitive biotype + and *n* = 2 cognitive biotype −; *χ*^2^ = 0.389, *P* = 0.533; Supplementary Table 4). The same was true for remitters (*n* = 3 and 1, respectively; *χ*^2^ = 0.019, *P* = 0.892; Supplementary Table 4). Across participants, the overall response rate was 25% and the remitter rate was 12.5%. This is in line with the National TMS Program, where MDD response and remission rates were 30.5% and 15.3%, respectively^27^.

### Consideration of potential contributing variables

We tested whether our results held when considering other potential contributing variables, including activation of the cognitive control circuit, motion during fMRI and reproducibility of performance inside and outside the scanner.

First, other than cognitive control, no differences in domains of cognition at baseline were noted between veterans in cognitive biotypes + and −. From the WebNeuro tasks, we analyzed verbal memory, working memory (digit span) and sustained attention (continuous performance test), and found that the performance of the two biotypes did not significantly differ in any of these (verbal memory, *t* = −0.404, *P* = 0.689, d.f. = 29.592, *d* = −0.148, 95% CI = [−0.787; 0.527]; working memory, *t* = 0.061, *P* = 0.952, d.f. = 26.321, *d* = 0.024, 95% CI = [−1.090; 1.157]; sustained attention, *t* = 0.433, *P* = 0.668, d.f. = 29.229, *d* = 0.160, 95% CI = [−0.397; 0.610]). They also did not differ in terms of symptom severity (Wilcoxon test statistic (*W*) = 201.5, *P* = 0.876), age (*t* = −0.747, *P* = 0.463, d.f. = 22.092, *d* = −0.318, 95% CI = [−14.385; 6.768]) or sex (*χ*^2^ = 0.188, *P* = 0.664).

Performance while conducting the cognitive control test outside the scanner was correlated with performance in the same task inside the scanner, establishing the reproducibility of performance measures (Spearman’s *ρ* = 0.313, *P* = 0.006).

The early improvement in cognitive control circuit connectivity following TMS that was dependent on pre-TMS stratification was still present when we accounted for activation of the cognitive control circuit as a covariate (*t* = 4.574, d.f. = 76.1, *d* = 1.049, 95% CI = [0.735; 1.868], *P* < 0.001; Supplementary Table 5) and the same was true for changes in cognitive control behavioral performance (*t* = 2.892, d.f. = 51.5, *d* = 0.806, 95% CI = [0.235; 1.298], *P* = 0.006; Supplementary Table 6). As before, depression severity decreased similarly in both biotypes (early treatment, *t* = −2.458, d.f. = 72.2, *d* = −0.578, 95% CI = [−3.06; −0.320], *P* = 0.016; post treatment, *t* = −1.996, d.f. = 95.1, *d* = 0.409, 95% CI = [−5.95; −0.017], *P* = 0.049; Supplementary Table 7).

Similarly, scanner motion (defined as the number of volumes having a framewise displacement of ≥0.3) did not change the significance of our findings when included as a covariate (early improvement in cognitive control circuit connectivity after TMS in cognitive biotype + veterans: *t* = 4.835, *P* < 0.001, d.f. = 73.1, *d* = 1.131, 95% CI = [0.777; 1.867]; early improvement in cognitive control behavioral performance in cognitive biotype + veterans: *t* = 3.057, *P* = 0.004, d.f. = 49.4, *d* = 0.870, 95% CI = [0.275; 1.331]; early improvement in depression severity regardless of cognitive biotype: *t* = −2.379, *P* = 0.020, d.f. = 68.5, *d* = −0.575, 95% CI = [−2.92; −0.257]; Supplementary Tables 8–10). In addition, motion did not show a significant effect in predicting changes in connectivity between the left dLPFC and dACC or changes in depression severity (all *P* > 0.05; Supplementary Tables 8 and 10). It did show a significant effect in predicting changes in Go–NoGo performance but as the addition of motion in the model did not change the significance of our predictor of interest (Biotype × TMS session), we did not investigate this further (Supplementary Table 9).

Furthermore, performance changes in aspects of cognition other than cognitive control following TMS were not related to changes in cognitive control circuit connectivity, thereby demonstrating that our findings were specific to cognitive control and not cognition in general (verbal memory, *r* = −0.077, *P* = 0.468, 95% CI = [−0.278; 0.131]; working memory, *r* = 0.051, *P* = 0.643, 95% CI = [−0.163; 0.260]; sustained attention, *r* = 0.017, *P* = 0.876 and 95% CI = [−0.191; 0.223]).

At baseline, cognitive biotype + veterans had worse behavioral cognitive control performance compared with the cognitive biotype − veterans (*t* = −2.127, *P* = 0.041, *d* = −0.759). After early TMS, the cognitive biotype + veterans showed improvements in connectivity (*t* = 4.784, *P* < 0.001, *d* = 1.114), which persisted post treatment (*t* = 3.347, *P* = 0.001, *d* = 0.675). Behavioral cognitive performance also improved (*t* = 3.862, *P* < 0.001, *d* = 1.075 and *t* = 3.177, *P* = 0.002, *d* = 0.767). There was no change in the cognitive biotype − veterans. Change in brain connectivity-mediated improvements in behavioral cognitive control performance (mediation effect = 0.140, *P* = 0.026). Depression symptoms improved for all veterans (*t* = −2.337, *P* = 0.021, *d* = −0.460).

## Discussion

We show that the biotype of an individual at baseline is important for determining whether they experience early improvements in their brain connectivity and cognitive function when treated with therapeutic TMS. Individuals designated as cognitive biotype +, with poor connectivity and cognitive control before commencing TMS, showed an early improvement in both of these measures after early TMS sessions and this improvement was maintained at the end of the therapeutic program. The magnitude of improvement in functional connectivity was directly correlated with improvement in behavioral performance as a function of their baseline biotype and the ‘dose’ of TMS they received, from the earlier to later number of sessions. In fact, change in brain connectivity mediated improvements in the behavioral capacity to control cognitive performance, a capacity highly relevant to how individuals manage the demands of day-to-day function. These findings highlight brain imaging of functional connectivity during cognitive control as a promising precision mental health approach with utility to identify individuals who are most likely to benefit early from TMS, potentially helping to alleviate longer-term disability.

Our findings elucidate the neurobiological underpinnings of how TMS may improve function in depression. It might do so in part by modulating the neural circuits involved in cognitive control—in particular, connectivity between the dLPFC and dACC and the therapeutic effects of TMS on the cognitive control circuit may depend on the extent of impairment in these circuits. In this study we stratified individuals according to the extent of impairment in dLPFC–dACC connectivity elicited during a cognitive control task relative to a healthy norm. Individuals with less connectivity were designated as cognitive biotype + and those with relatively intact connectivity as cognitive biotype −. Veterans in cognitive biotype + were distinguished by comparatively poorer performance on a behavioral test of cognitive control, verifying the connectivity-based stratification at baseline. This separation of the cognitive biotype group aligns with our previous work describing a cognitive dyscontrol biotype in MDD, characterized by cognitive deficits detectable by behavioral testing and specific functional impairment of the cognitive control circuit^11^. Our demonstration that therapeutic TMS mediated improvements in dLPFC–dACC connectivity and cognitive control performance specifically in the cognitive biotype + suggests that this circuit connectivity measure has promise as a novel stratification and TMS response biomarker for individuals experiencing cognitive impairment with depression. Use of connectivity biomarkers might help maximize the potential of TMS to ameliorate the cognitive impairments often associated with treatment-resistant depression, which contribute substantially to the disabling and chronic impact of this disorder. Given that the cognitive biotype + has been associated with a lower rate of response to antidepressant medication^11^, the use of a biomarker to help expedite these individuals to augmentation with TMS could improve function and limit chronic burden of illness.

Although there was a tight coupling between TMS-induced improvements in both dLPFC–dACC connectivity and cognitive control performance, relevant to functional outcomes, improvements in overall symptom severity were not dependent on baseline stratification. This finding raises the interesting possibility that therapeutic TMS might play a critical role in reconfiguring neural connectivity to improve objectively tested cognitive function, whereas other factors or neural circuits might underpin the improvement in self-reported symptoms. Clinical assessment of depression requires that symptoms cause notable impairment in social, occupational or other important areas of functioning, and cognitive capacity is tied tightly to these. Thus, future studies are needed to determine whether the improvement in behavioral performance in a cognitive control task following TMS that we detected is accompanied by an improvement in quality of life and everyday functioning rather than self-reported symptoms of depression.

Here we evaluated task-evoked functional brain connectivity as an imaging biomarker both for pretreatment stratification and for quantifying TMS response after early treatment as well as after treatment. Our results suggest that such early sampling of mechanistic imaging information may be an important novel approach for future precision studies of brain stimulation^15,28^. Methodologically, our findings extend previous research showing that resting-state connectivity involving the dLPFC and other regions of the ACC (such as the subgenual ACC) predicts overall symptom outcomes^29–31^ and increases post treatment in TMS symptom responders^32^. Although resting connectivity has helped identify symptom responders, task-evoked dLPFC connectivity may be particularly pertinent as a biomarker of functional improvements in patients with task-related cognitive impairments. In these patients the improvement of cognitive control performance may be of benefit above and beyond the improvement in depression symptoms, especially in light of evidence that cognitive impairments can persist despite symptom response^4,8^. Interestingly, dLPFC connectivity evoked by the same cognitive task as used in the present trial has also been identified as an important differential predictor of response to different types of antidepressant medications in depression^12^. Given that therapeutic TMS is currently given as an augmentation to ongoing pharmacotherapy, dLPFC connectivity has the potential to be a biomarker that helps identify the specific combination of TMS and medication to optimize functional improvements in depression.

Unfortunately, we can only speculate at the possible cellular effects of TMS within the described model, as cellular effects cannot be directly visualized in the human brain with the technology available at present. That caveat aside, because TMS works through local and distal effects (that is, localized depolarization of targeted neural populations coupled with modulation of large-scale neural networks), it would be reasonable to hypothesize that TMS is able to differentially impact the underlying etiology related to the cognitive biotype. One can imagine a pathological homeostasis exists in the cognitive biotype such that regional activity is insufficient to meet the cognitive needs of a patient and that TMS can disrupt this pathology acutely, and with longer-term administration induce a neuroplastic effect that yields longer-term disruption of the underlying pathology. This is supported by studies of in vitro and in vivo models that have shown that TMS increases the release of growth factors and changes to neurotransmitter levels^33–36^. However, further research is required to understand the complex molecular signaling cascades and circuitry-based alterations that occur following rTMS.

Our results should be interpreted in light of certain limitations. The observed changes in functional connectivity and cognitive performance were assessed in the context of a naturalistic TMS treatment regime of veterans and using self-reported symptom measurement. Further research is required to confirm these findings in broader clinical settings. In addition, the changes we observed were detected at the end of TMS treatment but we did not assess whether they persisted beyond that. Future studies could examine whether these changes are sustained in the absence of TMS stimulation. We acknowledge that to define our cognitive biotype, we applied a cutoff on continuous baseline left dLPFC–dACC functional connectivity. Our approach was motivated by the clinical need for a stratification that would identify patients with functional impairment of the cognitive controls circuit and our rationale for choosing the mean of a healthy sample as the cutoff was based on ease of interpretation. Still, we acknowledge that binarizing a continuous variable comes at the price of loss of information. Moreover, the fact that we applied only one cutoff strategy is a limitation and future studies could investigate different cutoffs or try to identify an optimal cutoff for a given purpose (for example, treatment-response prediction). Furthermore, when separating veterans by biotype, the resulting groups were quite small. Future studies should apply our biotyping strategy to larger samples. The interplay of left dLPFC activation and connectivity after TMS is still unclear. Interestingly, when we added changes in left dLPFC activation to our models as a covariate, they did not significantly predict changes in cognition or symptoms, which suggests that although dLPFC activation might be a stratification biomarker of a cognitive biotype of depression^11^, dLPFC–dACC connectivity might rather be a response biomarker. In addition, the specific mechanisms through which TMS brings about these neural changes and cognitive improvements in a specific subtype of depression are not entirely clear and warrant further investigation.

In conclusion, this study shows that dLPFC–dACC connectivity measured during a cognitive control task is a promising biomarker for the stratification of a cognitive biotype of depression, which might be particularly suited to TMS treatment. This connectivity measure also offers a potential response biomarker for quantifying the trajectory of improvement in the cognitive biotype. These results have particular significance for the types of depression that are usually less responsive to pharmacotherapy and that dramatically impair function and quality of life. Our findings pave the way for delivering TMS within a precision mental health approach in which imaging biomarkers could be used for earlier identification of individuals that may be likely to benefit from TMS and an explanation for why.

## Methods

### Study design

The BiomarkerS for Transcranial Magnetic Stimulation Antidepressant Response to Treatment-fMRI (B-SMART-fMRI) study followed a pragmatic precision medicine design. Veterans with MDD who were eligible for TMS in a neuromodulation clinic were offered participation. Biomarker measures of fMRI, cognitive behavioral performance and clinical symptoms were assessed at pretreatment baseline (within 72 h before their first TMS treatment), early in the treatment process (within 24 h of their fifth TMS treatment) and post treatment (within 72 h of their thirtieth TMS treatment). The study design flow is explained in Supplementary Fig. 9 and the original protocol^23^. The trial was coordinated through Stanford University and veterans were assessed at one of four clinics participating in the National TMS Clinical program^27^. The four clinics were located at four Veterans Affairs sites (each with affiliated universities): Palo Alto (Stanford University), White River Junction (Dartmouth College), Providence (Brown University) and Minneapolis (University of Minnesota). Each collaborating Veterans Affairs site is working closely with their local University to obtain brain scans. The study received Institutional Review Board (IRB) approval for all corresponding institutions and appropriate guidelines were followed (52695 for Stanford University, 1594812–6 for Minneapolis Veterans Affairs Health Care System, 1633756–11 for Providence Veterans Affairs Health Care System and 1531845–1 for Veteran’s IRB of Northern New England). This trial was registered on ClinicalTrials.gov under NCT04663481.

The partnership with the TMS National Clinical program enabled this trial to be undertaken in real world clinical settings. We also controlled the interpretation of clinical outcomes by comparing clinical symptom outcomes for participants who completed biomarker assessments to those for the broader, generalizable sample of veterans in the national program.

Data in the present analysis are from the planned wave 1 midpoint of the trial.

### Samples

The study aims to recruit 125 veterans (with an anticipated dropout rate of 20%) with treatment-resistant depression from the Veterans Administration Clinical TMS Program. Here, we focus on wave 1, the first 50% of samples.

Veterans completed an initial pre-screening to assess eligibility criteria. Inclusion criteria were: ≥18 yr old, meet the diagnostic criteria for MDD according to the *Diagnostic and Statistical Manual of Mental Disorders* (DSM-5)^37^ as confirmed by the TMS physician, demonstrate pharmacoresistance by failing to respond to at least one antidepressant trial of adequate dose and duration, be capable of obtaining a motor threshold measurement before treatment, have stable medical conditions, be able to maintain their current medication regimen throughout the treatment period, be able to participate in a daily treatment regimen and possess the ability to read, understand and voluntarily sign the informed consent form. Exclusion criteria included: a history of seizure disorder; the presence of structural or neurological abnormalities near the treatment site; a history of brain surgery; the presence of a pacemaker or a non-MRI-compatible medical infusion device; a history of traumatic brain injury within 60 ds of treatment initiation, severe or uncontrolled alcohol or substance use disorders; active withdrawal from alcohol or substances; the presence of an implanted device or metal in the head; severe impairment of vision, hearing or hand movement that would interfere with assessments or protocol adherence; a lifetime history of bipolar I disorder; inability to speak, read or understand English; plans to relocate outside the study area during the study period and exclusion at the discretion of the clinician or investigator due to clinical safety or protocol adherence concerns. The participants were compensated for their time.

A total of 55 participants signed the informed consent form, 43 of which completed fMRI at the planned wave 1 of the trial (mean age, 48 yr; 83.7% male and 16.3% female; Table 1 and Supplementary Fig. 6 for CONSORT chart). Of these, 33 completed all clinical follow-ups. All available data were used for our statistical analyses, including that of veterans with some incomplete time points.

In addition to these clinical samples, we used previously collected normative samples of 144 healthy controls (mean age, 32.16 yr; 50.7% male and 49.3% female; Supplementary Table 11) to quantify our imaging measure of interest relative to a reference norm following a validated protocol^38^.

### TMS protocol

Veterans received TMS treatment as part of their regular clinical care within the VA clinical TMS program. Thus, we followed all the procedures of the clinics within the program^27^. In summary, treatment was delivered using Magstim devices with figure-of-eight coil (The Magstim Company Ltd). Head dimensions were measured to localize the approximate area on the motor cortex controlling the left thumb using the Beam F3 method^39^. Motor threshold was generally identified using the parameter estimation by sequential testing program and reported as a percentage of maximal machine output^40^. Parameter estimation by sequential testing is an automated approach to establish resting motor threshold (available at clinicalresearcher.org). Briefly, TMS clinicians enter a starting energy level, monitor for movement in the contralateral hand and depending on a binary response (yes/no) about the presence or absence of movement in this hand, the algorithm will suggest the next intensity to assess. After a certain number of responses, it will converge on the resting motor threshold. The target of the stimulation was the left dLPFC. Further details on the treatment protocol are available in refs. 23,27.

### Neuroimaging

Neuroimages were acquired using 3 T GE Healthcare Discovery MR750 UHP (at Palo Alto) and 3 T Siemens Medical Solutions Magnetom Prisma Fit (at Minnesota, Providence and White River Junction) scanners. Details on all imaging data collected have been previously published^23^. The parameters of the sequences relevant to the current analysis are presented in the Supplementary Methods.

### Task fMRI design.

Task fMRI data were collected during a Go–NoGo paradigm to assess response inhibition and cognitive control (Fig. 1). This task is well normed across nine decades and has sound test–retest reliability^41^. It has been shown to robustly elicit inhibition errors in MDD and post-traumatic stress disorder^12,42^. In the Go–NoGo task, veterans respond via button press as quickly and accurately as possible to Go stimuli (the word ‘press’ in green) and withhold responses to NoGo stimuli (the word ‘press’ in red). There are 180 Go and 60 NoGo stimuli (ratio 3:1), presented pseudorandomly with a duration of 500 ms and jittered interstimulus interval of 750 ± 50 ms. Behavior was assessed with Commission NoGo errors and Omission Go errors.

#### Preprocessing.

The fMRI data were preprocessed and analyzed using SPM version 8 (www.fil.ion.ucl.ac.uk/spm) and FSL version 6 with default parameters, unless otherwise specified. Motion correction was performed by realigning and unwarping the fMRI images to the first image of each task run. For normalization to stereotactic Montreal Neurological Institute (MNI) space, the T1-weighted data were normalized to MNI space using the FMRIB nonlinear registration tool, and the fMRI EPI data were co-registered to the T1 data using the FMRIB linear registration tool^43^. Normalization warps from these two steps were stored for use in functional-to-standard space transformations.

#### Task analysis.

To compute task-related activations, the onset times of Go and NoGo stimuli were convolved with a canonical hemodynamic response function, and entered in a general linear model analysis as regressors of interest and the six realignment parameters as regressors of no interest. A high-pass filter with a cutoff period of 128 s was applied.

The left dLPFC and dACC were defined as described previously^38^. Briefly, a search was conducted for ‘cognitive control’ in the meta-analytic database Neurosynth^44^, the resulting map was thresholded at positive false discovery rate < 0.01, peak coordinates of the resulting clusters were identified and voxels at a maximum of 10 mm from these peaks were used to generate regions of interest. The peak for left dLPFC was at MNI coordinates −44, 6, 32 and that for dACC was at MNI coordinates 0, 18, 46. To quantify task-based functional connectivity, we computed psychophysiological interactions using the left dLPFC as a seed region. We calculated the first eigenvariate of the left dLPFC time series and fit a whole-brain general linear model as described earlier, which consisted of the psychological variable of interest (NoGo>Go contrast), physiological variable (left dLPFC time course) as well as the interaction between psychological and physiological variables (psychophysiological interaction effect of interest). Finally, the mean values of the psychophysiological interaction contrast were extracted from the left dLPFC.

#### Correction for scanner effects.

Before aggregating data collected on different scanners, we removed the potential confounding effect of between-scanner variability using ComBat^45–47^, an established method that uses an empirical Bayesian framework to remove batch effects.

#### Referencing to a healthy norm.

Left dLPFC–dACC NoGo>Go connectivity was expressed in s.d. units relative to the mean and s.d. of a sample of healthy controls undergoing the same task (*z*-scores). Our approach to quantify participant-level measures of brain circuit dysfunction relative to a healthy norm is built on our previous work, in which we established this procedure^38^. Subsequent analyses were conducted only on the left dLPFC–dACC NoGo>Go connectivity of veterans with treatment-resistant depression.

#### Cognitive control performance.

Computerized tests of cognitive control performance were collected at baseline, after the fifth TMS treatment and post TMS treatment on a computer using the cognitive test battery WebNeuro. Psychometric properties for WebNeuro have been established and include norms, construct validity, validation against traditional neuropsychological tests evaluating equivalent functions, test–retest reliability and consistency across cultures^48–53^.

Here the main task of interest was a Go–NoGo task in which a word (press) is frequently presented in the color green (Go) and infrequently in the color red (NoGo). Veterans were asked to respond with a keypress when the word was presented in green and inhibit a keypress when it was presented in red. Inhibition was assessed with omission of keypress responses when the word ‘press’ was red. Behavior was assessed in terms of Commission NoGo errors and Omission Go errors. The sum of Commission NoGo errors and Omission Go errors expressed relative to an age- and sex-matched healthy norm provided by WebNeuro was used for statistical analyses as the measure of cognitive control performance. Note that the Go–NoGo task is the same task that was performed inside the scanner during fMRI (described earlier).

We also obtained overall performance measures of verbal memory, working memory and sustained attention from WebNeuro and used these to test whether our behavioral performance results were specific to cognitive control (described below). The overall performance measure for each task was the mean of all the normed measures available in that task.

### Depression severity

Depression severity was assessed at baseline, after early TMS treatment, and post TMS treatment using the QIDS total^54^. A participant was considered a responder to the treatment if they showed a decrease of QIDS total of more than 50% from baseline and they were considered a remitter if their post-treatment QIDS total was ≤5.

### Statistical analysis

All statistical analyses were conducted in R version 4.1.3 using R Studio Version 2023.06.2+561. First, we divided the treatment-resistant depression samples into two biotypes based on their baseline left dLPFC–dACC NoGo>Go connectivity. Given that zero represented the mean of our healthy reference sample, the cognitive biotype was defined as participants who have hypoconnected left dLPFC–dACC NoGo>Go connectivity of <0 (*n* = 26). We labeled these participants as cognitive biotype +. By contrast, those not in the cognitive biotype group were defined as participants who have relatively intact connectivity of ≥0 (*n* = 17). We labeled these participants as cognitive biotype −. This biotype variable was then used in subsequent models testing for the effects of baseline connectivity and TMS treatment on changes in connectivity, cognitive performance and symptom severity in time.

### Effect of TMS on cognitive control circuit connectivity

We ran a linear mixed-effects model in which our pretreatment stratification factor was cognitive biotype, our dependent measure was change in brain connectivity across TMS sessions and our focal effect of interest was the interaction of biotype and TMS session. In this model fixed effects were biotype (between participants, cognitive biotype + or −) and TMS session (repeated measure within participants, baseline, early treatment and post treatment) and we covaried for the continuous measure of days from baseline on the scan date. We tested the significance of the interaction using an *F*-test and used *t*-tests for planned contrasts between TMS sessions for each biotype.

This model implements the analytic strategy in our protocol^23^ and is designed to test the hypothesis that change in connectivity induced by TMS will be a function of pretreatment impairment such that when a binary grouping is used to define connectivity impairment at baseline, the trajectory of change in TMS-related connectivity will differ between veterans with hypoconnectivity (cognitive biotype +) and veterans with relatively intact connectivity (cognitive biotype −).

We took several analytic considerations into account. These included the emphasis on change in connectivity as our dependent outcome measure, a difference score value that is not fully determined by the single pretreatment raw value of connectivity. We also verified that TMS session and days from baseline were not colinear when entered in a linear model predicting difference in brain connectivity from baseline (variance inflation factors, 3.84 and 3.83, respectively). Furthermore, we incorporated random intercepts for each participant to account for any similarities between measurements taken from the same participant.

### Effect of TMS on cognitive control performance

First, we compared cognitive control performance between veterans in the cognitive biotype + and − groups using a *t*-test. We then ran a linear mixed-effects model, with fixed effects of pretreatment biotype (cognitive biotype + or −), TMS session (repeated measure within participants, baseline, early treatment and post treatment) and the TMS-induced change in cognitive control performance as our dependent variable. We covaried days from baseline on the cognitive testing date (continuous) and incorporated random intercepts for each participant to account for any similarities between measurements taken from the same individual. Our primary effect of interest was the biotype by TMS session interaction and we tested its significance using an *F*-test and *t*-tests for planned contrasts comparing between sessions for each biotype.

To further link the changes in left dLPFC–dACC NoGo>Go connectivity from baseline and those in cognitive control performance across all sessions, we tested whether these were correlated using a Pearson correlation.

### Mediation analysis of connectivity and behavior

To determine whether the changes in cognitive control performance were driven by those in functional connectivity, we built a causal mediation model using the ‘mediation’ package in R. In this model the independent variable was biotype (factor: cognitive biotype + or −), the mediator variable was change in left dLPFC–dACC NoGo>Go connectivity from baseline and the dependent variable was change in cognitive control performance from baseline. We considered average causal mediation effects and average direct effects to be significant if *P* < 0.05.

Finally, we demonstrated generalizability of our cognitive control performance measure by conducting a Spearman correlation between total Go–NoGo errors in-scanner and out-of-scanner across all sessions.

### Effect of TMS on depression severity

We ran a linear mixed-effects model using the difference in depression severity (measured by total QIDS) from baseline as our dependent variable. As fixed effects in our model, we included biotype (factor: cognitive biotype + or −), TMS session (factor: baseline, early treatment and post treatment) and their interaction, along with days from baseline on the symptom assessment date (continuous). We also incorporated random intercepts for each participant to account for any similarities between measurements taken from the same participant. Our effect of interest was the Biotype * TMS session interaction, and we tested its significance using an *F*-test. On finding a significant interaction, we followed up the result with post-hoc *t*-tests comparing the two biotypes at each TMS session.

We also tested whether veterans in the cognitive biotype + had a higher rate of response or remission using a *χ*^2^ test.

### Left dLPFC activation, motion and other cognitive tests

To test whether the NoGo>Go activation of the left dLPFC (as opposed to the connectivity) influenced our results, all linear mixed models outlined above were rerun with NoGo>Go activation of the left dLPFC as a covariate.

Similarly, we tested whether in-scanner motion influenced our results by including the number of volumes with a framewise displacement of ≥0.3 (ref. 55) for each session and participant as a covariate in all linear mixed models.

Finally, to test whether our behavioral performance results were specific to cognitive control, we compared the overall performance in WebNeuro tests measuring verbal memory, working memory and sustained attention of veterans in the cognitive biotype + and − groups. We also calculated the Pearson’s correlation between changes in left dLPFC–dACC NoGo>Go connectivity from baseline and those in performance in these tasks across all sessions.

### Reporting summary

Further information on research design is available in the Nature Portfolio Reporting Summary linked to this article.

## Supplementary Material

Supplementary material

## Figures and Tables

**Table 1 |:** Participant characteristics

	Cognitive biotype + (*n* = 26)	Cognitive biotype - (*n* = 17)
Age (yr; mean, s.d.)	47.08 (11.38)	48.76 (15.50)
Race, *n* (%)		
White	21 (80.8%)	14 (82.3%)
Black	1 (3.8%)	1 (5.9%)
Asian	2 (7.7%)	0
Multiracial	2 (7.7%)	1 (5.9%)
Other	0	1 (5.9%)
Ethnicity, *n* (%)		
Hispanic	0	1 (5.9%)
Not Hispanic	26 (100%)	16 (94.1%)
Gender, *n* (%)		
Male	23 (88.5%)	13 (76.5%)
Female	3 (11.5%)	4 (23.5%)
Highest education, *n* (%)		
High school/GED	7 (26.9%)	2 (11.8%)
Some college	4 (15.4%)	4 (23.5%)
Associates	4 (15.4%)	1 (5.9%)
Bachelor's degree	7 (26.9%)	7 (41.1%)
Master's degree	4 (15.4%)	3 (17.7%)
Sites, *n* (%)		
Palo Alto	7 (26.9%)	5 (29.5%)
Providence	8 (30.8%)	6 (35.3%)
Minnesota	9 (34.6%)	3 (17.6%)
White River Junction	2 (7.7%)	3 (17.6%)
Diagnoses, *n* (%)		
Post-traumatic stress disorder	21 (80.8%)	10 (58.8%)
Substance use disorder	3 (11.5%)	1 (5.9%)
Alcohol use disorder	3 (11.5%)	2 (11.8%)
Anxiety disorders	21 (80.8%)	13 (76.5%)
Obsessive compulsive disorder	2 (7.7%)	2 (11.8%)
Attention deficit and hyperactivity disorder	2 (7.7%)	3 (17.6%)
Other	0	3 (17.6%)

GED, General Educational Development Test.

## Data Availability

Data will be shared through the NIMH Data Archive in accordance with the funding requirements following study completion (collection 3376).
