# Supplementary material for: A cognitive neural circuit biotype of depression showing functional and behavioral improvement after transcranial magnetic stimulation in the B-SMART-fMRI trial
Source: Nat Ment Health. Author manuscript; Available in PMC 2025 Feb 5. (PMC11798407; doi:10.1038/s44220-024-00271-9)
Supplement: Supplementary material [file NIHMS2047876-supplement-Supplementary_material.pdf]

nature mental health

Supplementary information https://doi.org/10.1038/s44220-024-00271-9

A cognitive neural circuit biotype of depression showing functional and behavioral improvement after transcranial magnetic stimulation in the B-SMART-fMRI trial

In the format provided by the authors and unedited

Table of Contents

Supplementary Methods 2

MRI sequence parameters 2

Supplementary Figures 3

Supplementary Figure 1: Changes in cognitive control circuit connectivity following TMS. 4

Supplementary Figure 2: Changes in behavior following TMS. 5

Supplementary Figure 3: Change in clinical severity changes following TMS. 6

Supplementary Figure 4: Plot of severity following TMS for each symptom. 7

Supplementary Figure 5: Flow Diagram of Study Procedures. 8

Supplementary Figure 6: The Consolidated Standards of Reporting Trials (CONSORT) diagram showing data available for the current study at each time points and reasons for exclusion of participants. 9

Supplementary Tables 10

Supplementary Table 1: Results of the mixed linear model predicting change in connectivity between the left dlPFC and dACC. 11

Supplementary Table 2: Results of the mixed linear model predicting change in Go-NoGo performance. 12

Supplementary Table 3: Results of the mixed linear model predicting change in depression severity measured by QIDS total. 13

Supplementary Table 4: Responders and remitters post-treatment. 14

Supplementary Table 5: Results of the mixed linear model predicting change in connectivity between the left dlPFC and dACC accounting for left dlPFC activation. 15

Supplementary Table 6: Results of the mixed linear model predicting change in Go-NoGo performance accounting for left dlPFC activation. 16

Supplementary Table 7: Results of the mixed linear model predicting change in depression severity measured by QIDS total accounting for left dlPFC activation. 17

Supplementary Table 8: Results of the mixed linear model predicting change in connectivity between the left dlPFC and dACC accounting for motion. 18

Supplementary Table 9: Results the mixed linear model predicting change in Go-NoGo performance accounting for motion. 19

Supplementary Table 10: Results of the mixed linear model predicting change in depression severity measured by QIDS total accounting for motion. 20

Supplementary Table 11: Healthy control sample characteristics. 21

Supplementary Methods

MRI sequence parameters

Palo Alto:

- Task fMRI: TE = 27.50 ms, TR = 2 s, FA = 77, acquisition time = 5:08 (Go-NoGo), field of view = 222 ×222mm, 3D matrix size = 74 ×74 ×45, slice orientation = axial, angulation to AC-PC line, phase encoding = PA, number of volumes = 151, voxel size = 3 mm isotropic.
- T1-weighted anatomical: TE = 3.8 ms, TR = 3 s, FA = 8, field of view = 256 ×256mm, 3D matrix size = 320 × 320 × 230, slice orientation = sagittal, angulation to AC-PC line, motion correction = PROMO, voxel size = 0.8 mm isotropic; parallel imaging technique = GRAPPA/ARC.

Minnesota:

- Task fMRI: TE = 27.0 ms, TR = 2 s, FA = 77, acquisition time = 5:12 (Go-NoGo), field of view = 222 ×222mm, 3D matrix size = 74 ×74 ×45, slice orientation = axial, angulation to AC-PC line, phase encoding = AP, number of volumes = 151, voxel size = 3 mm isotropic.
- T1-weighted anatomical: TE = 3.77 ms, TR = 2.8 s, FA = 8, field of view = 256 ×256mm, 3D matrix size = 320 × 320 × 230, slice orientation = sagittal, angulation to AC-PC line, motion correction = PROMO, voxel size = 0.8 mm isotropic; parallel imaging technique = GRAPPA/ARC.

Providence:

- Task fMRI: TE = 27.0 ms, TR = 2 s, FA = 77, acquisition time = 5:06 (Go-NoGo), field of view = 222 ×222mm, 3D matrix size = 74 ×74 ×45, slice orientation = axial, angulation to AC-PC line, phase encoding = PA, number of volumes = 151, voxel size = 3 mm isotropic.
- T1-weighted anatomical: TE = 3.77 ms, TR = 2.8 s, FA = 8, field of view = 256 ×256mm, 3D matrix size = 320 × 320 × 230, slice orientation = sagittal, angulation to AC-PC line, motion correction = PROMO, voxel size = 0.8 mm isotropic; parallel imaging technique = GRAPPA/ARC.

White River Junction:

- Task fMRI: TE = 27.0 ms, TR = 2 s, FA = 77, acquisition time = 5:06 (Go-NoGo), field of view = 222 ×222mm, 3D matrix size = 74 ×74 ×45, slice orientation = axial, angulation to AC-PC line, phase encoding = AP, number of volumes = 151, voxel size = 3 mm isotropic.
- T1-weighted anatomical: TE = 3.77 ms, TR = 2.8 s, FA = 8, field of view = 256 ×256mm, 3D matrix size = 320 × 320 × 230, slice orientation = sagittal, angulation to AC-PC line, motion correction = PROMO, voxel size = 0.8 mm isotropic; parallel imaging technique = GRAPPA/ARC.

Supplementary Figures

Supplementary Figure 1: Changes in cognitive control circuit connectivity following TMS.

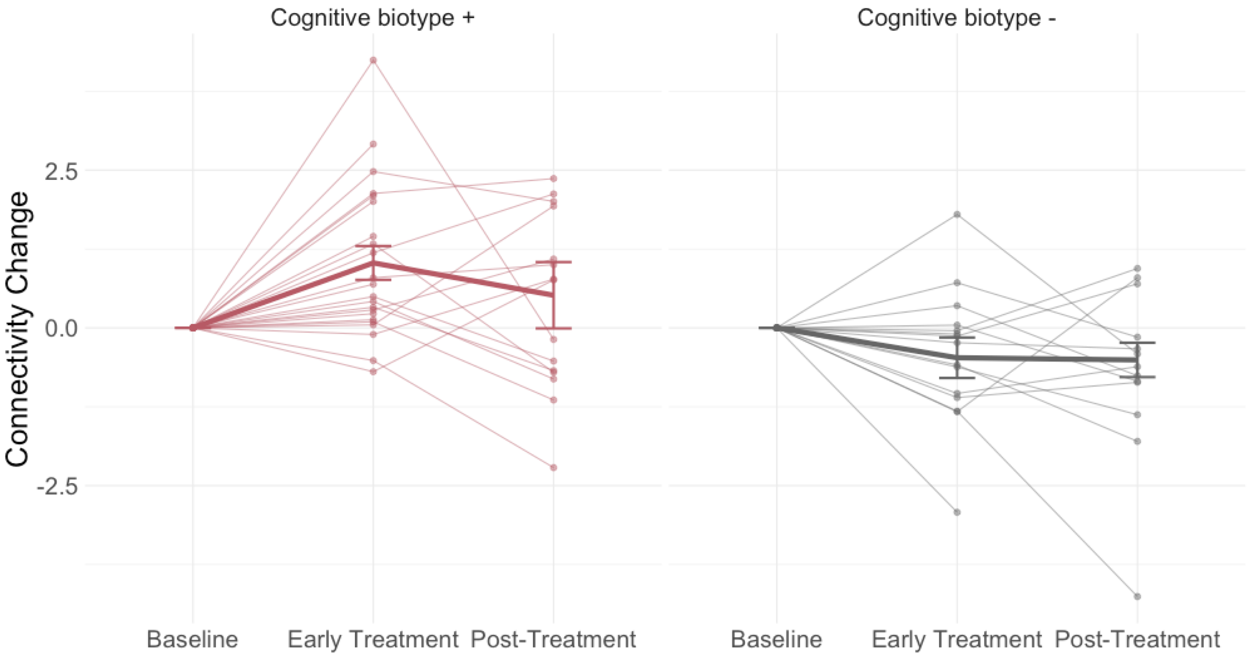

We show as thick lines the mean of changes from baseline of left dlPFC-dACC NoGo > Go connectivity of each biotype and the standard error of the mean as whiskers for each session, along with data from each patient (dots and faint lines). Note that in the main analyses cognitive control performance change was adjusted for days from baseline on the scan date and subject using a linear mixed-effects model. Here, we show the raw values with no adjustment. The cognitive biotype was defined by participants who have hypo-connected Left dlPFC-dACC NoGo > Go connectivity, less than 0 (N=26). We label these participants as "cognitive biotype +". By contrast, those not in the cognitive biotype were defined by participants who have relatively intact connectivity, greater or equal to 0 (N=17). We label these participants as "cognitive biotype -".

Abbreviations: dACC=dorsal anterior cingulate cortex, dlPFC=dorsolateral prefrontal cortex, TMS=therapeutic transcranial magnetic stimulation.

Supplementary Figure 2: Changes in behavior following TMS.

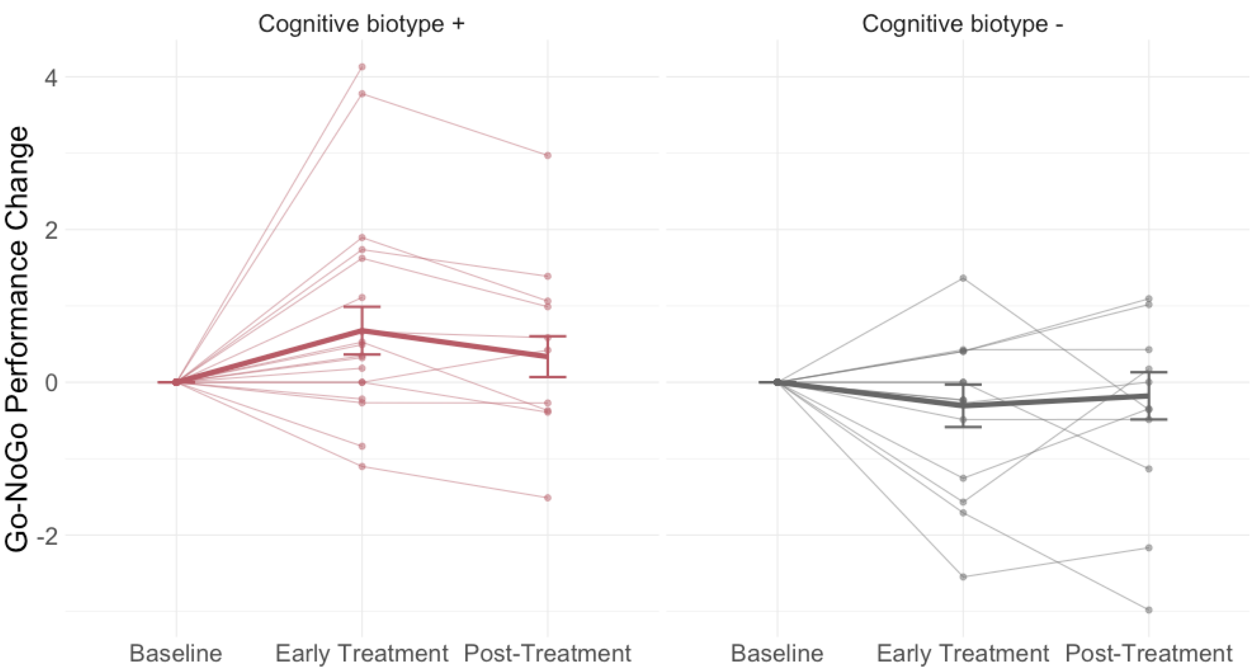

We show as thick lines the mean of changes from baseline of cognitive control performance of each biotype and the standard error of the mean as whiskers for each session, along with data from each patient (dots and faint lines). Note that in the main analyses cognitive control performance change was adjusted for days from baseline on the scan date and subject using a linear mixed-effects model. Here, we show the raw values with no adjustment. The cognitive biotype was defined by participants who have hypo-connected Left dlPFC-dACC NoGo > Go connectivity, less than 0 (N=26). We label these participants as "cognitive biotype +". By contrast, those not in the cognitive biotype were defined by participants who have relatively intact connectivity, greater or equal to 0 (N=17). We label these participants as "cognitive biotype -".

Abbreviations: dACC=dorsal anterior cingulate cortex, dlPFC=dorsolateral prefrontal cortex, TMS=therapeutic transcranial magnetic stimulation.

Supplementary Figure 3: Change in clinical severity changes following TMS.

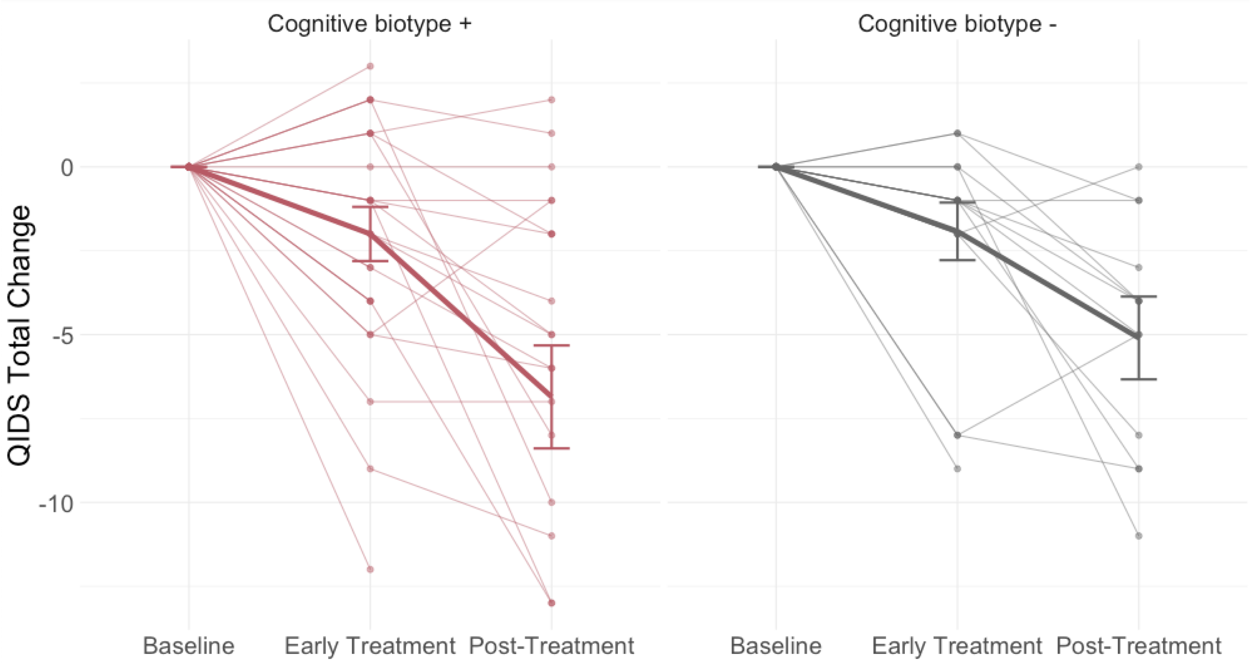

We show as thick lines the mean of changes from baseline of clinical depression severity (QIDS total score) of each biotype and the standard error of the mean as whiskers for each session, along with data from each patient (dots and faint lines). Note that in the main analyses clinical severity change was adjusted for days from baseline on the scan date and subject using a linear mixed-effects model. Here, we show the raw values with no adjustment. The cognitive biotype was defined by participants who have hypo-connected Left dlPFC-dACC NoGo > Go connectivity, less than 0 (N=26). We label these participants as "cognitive biotype +". By contrast, those not in the cognitive biotype were defined by participants who have relatively intact connectivity, greater or equal to 0 (N=17). We label these participants as "cognitive biotype -".

Abbreviations: dACC=dorsal anterior cingulate cortex, dlPFC=dorsolateral prefrontal cortex, QIDS=Quick Inventory of Depression Symptoms, TMS=therapeutic transcranial magnetic stimulation.

Supplementary Figure 4: Plot of severity following TMS for each symptom.

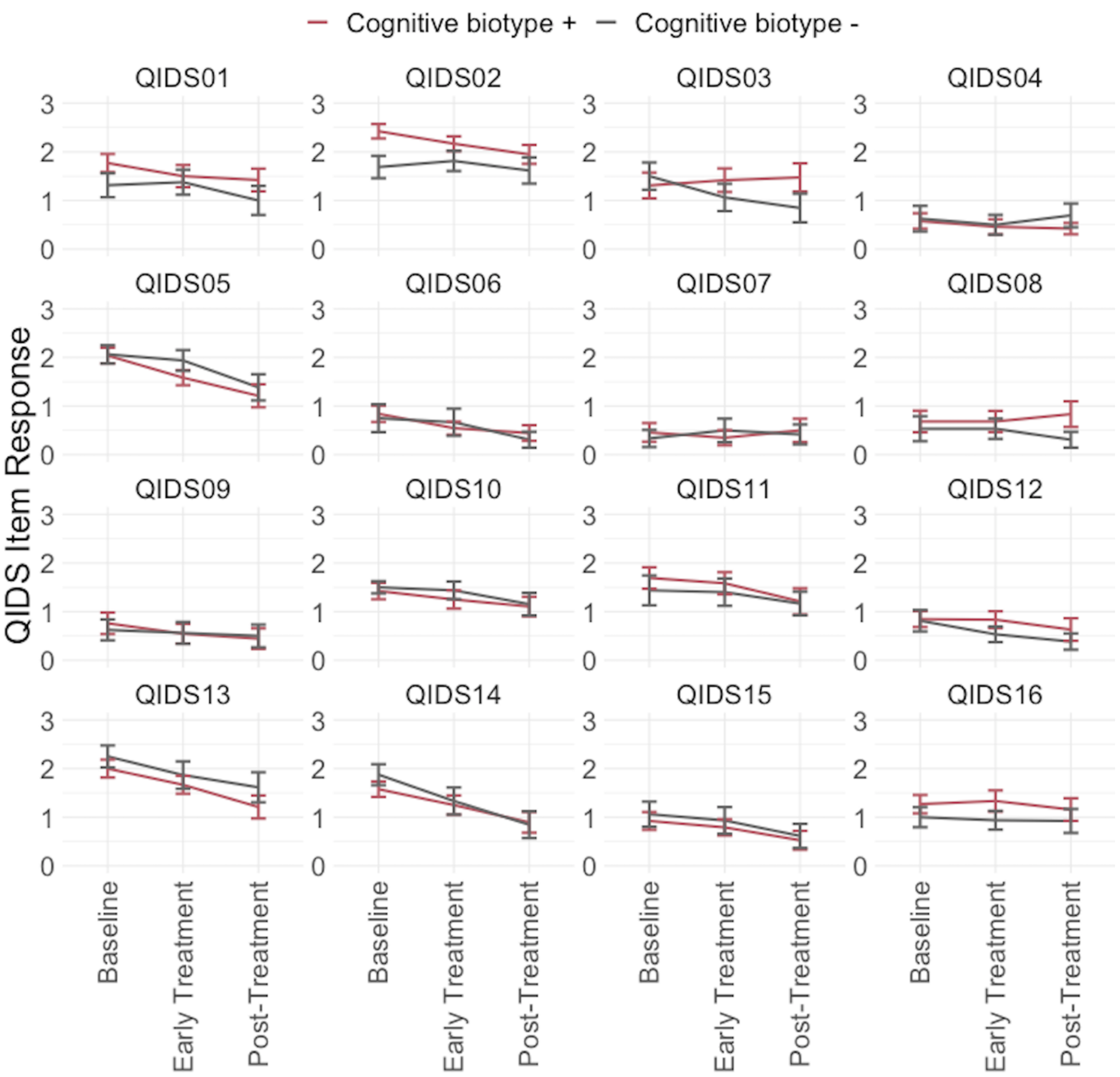

We show as thick lines the mean of clinical severity for each session for each QIDS item of each biotype and the standard error of the mean as whiskers. The cognitive biotype was defined by participants who have hypo-connected Left dlPFC-dACC NoGo > Go connectivity, less than 0 (N=26). We label these participants as "cognitive biotype +". By contrast, those not in the cognitive biotype were defined by participants who have relatively intact connectivity, greater or equal to 0 (N=17). We label these participants as "cognitive biotype -".
Abbreviations: dACC=dorsal anterior cingulate cortex, dlPFC=dorsolateral prefrontal cortex, QIDS=Quick Inventory of Depression Symptoms, TMS=therapeutic transcranial magnetic stimulation.

Supplementary Figure 5: Flow Diagram of Study Procedures.

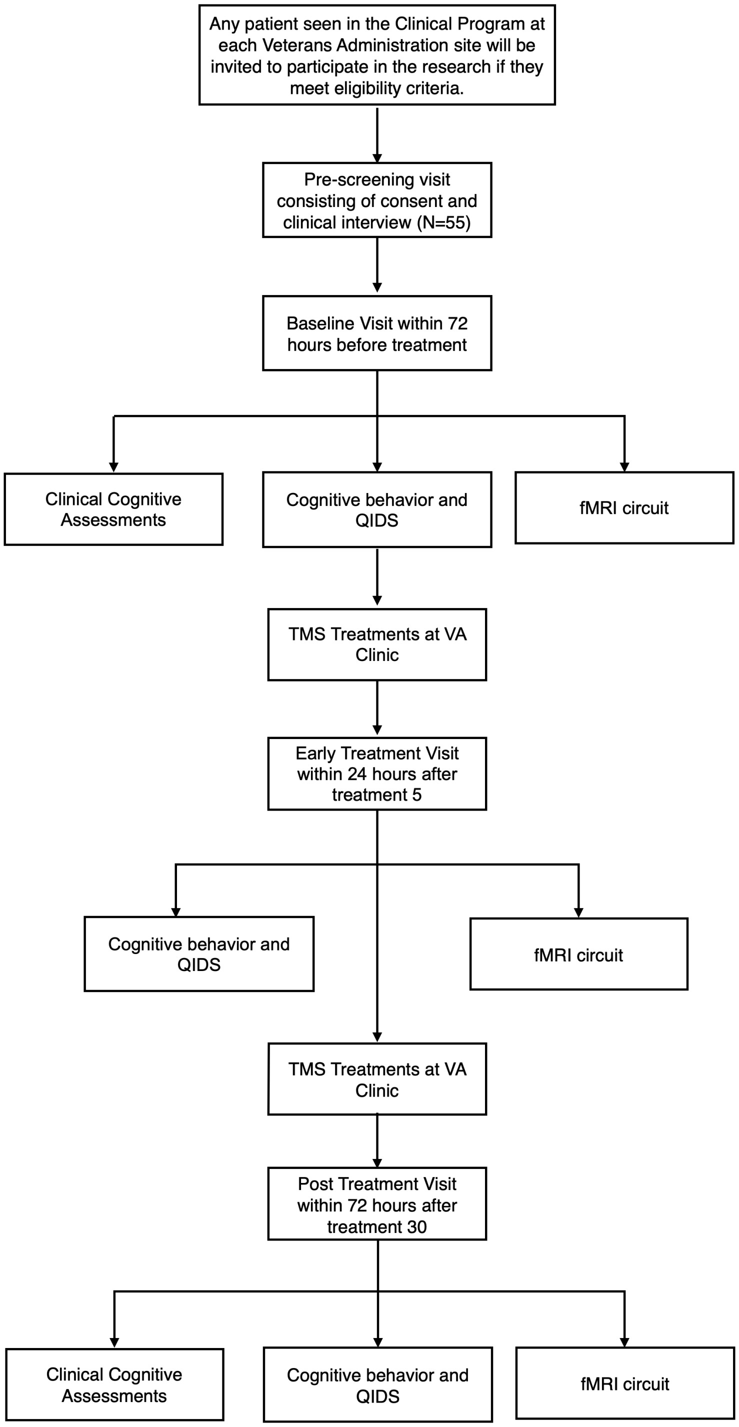

Abbreviations: QIDS=quick inventory of depressive symptomatology, TMS=therapeutic transcranial magnetic stimulation.

Supplementary Figure 6: The Consolidated Standards of Reporting Trials (CONSORT) diagram showing data available for the current study at each time points and reasons for exclusion of participants.

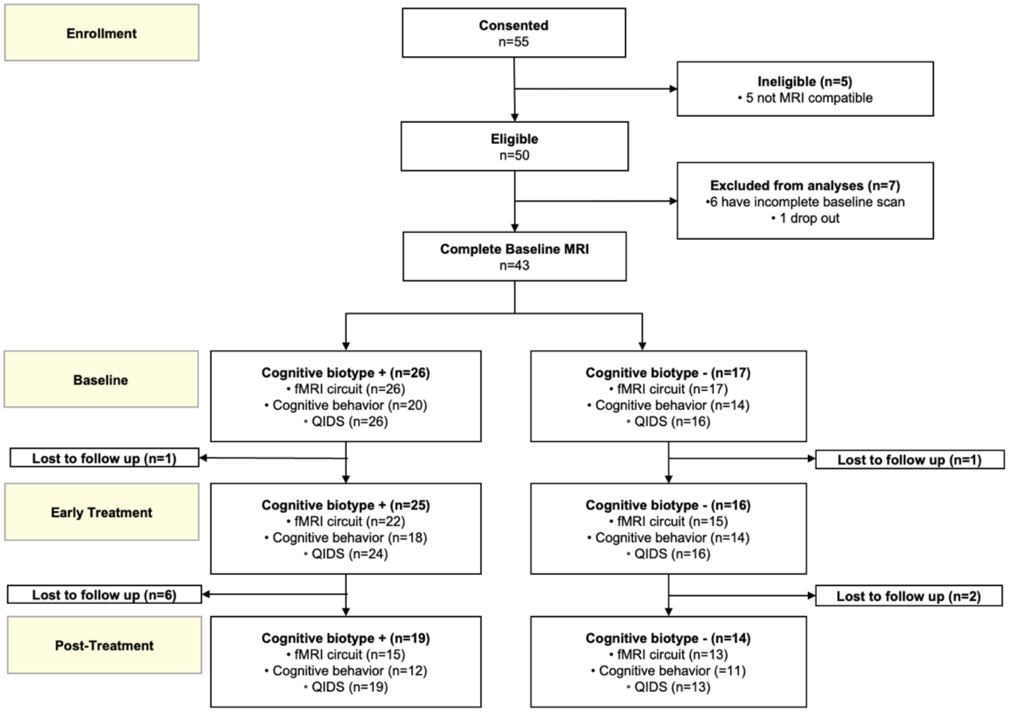

The cognitive biotype was defined by participants who have hypo-connected Left dlPFC-dACC NoGo > Go connectivity, less than 0 (N=26). We label these participants as "cognitive biotype +". By contrast, those not in the cognitive biotype were defined by participants who have relatively intact connectivity, greater or equal to 0 (N=17). We label these participants as "cognitive biotype -".
Abbreviations: dACC=dorsal anterior cingulate cortex, dlPFC=dorsolateral prefrontal cortex, MRI=magnetic resonance imaging, QIDS=quick inventory of depression symptoms, TMS=therapeutic transcranial magnetic stimulation.

Supplementary Tables

Supplementary Table 1: Results of the mixed linear model predicting change in connectivity between the left dlPFC and dACC.
The cognitive biotype was defined by participants who have hypo-connected Left dlPFC-dACC NoGo > Go connectivity, less than 0 (N=26). We label these participants as "cognitive biotype +". By contrast, those not in the cognitive biotype were defined by participants who have relatively intact connectivity, greater or equal to 0 (N=17). We label these participants as "cognitive biotype -". Time refers to days from the baseline session on the day of the dependent variable assessment. All statistical tests were two-sided and not adjusted for multiple comparisons.
Abbreviations: CI=confidence interval, dACC=dorsal anterior cingulate cortex, DF=degrees of freedom, dlPFC=dorsolateral prefrontal cortex, TMS=transcranial magnetic stimulation.

**Model effects**						
Effect	Sum Squares	Mean Squares	DF	Errors DF	F	p
Cognitive biotype – and +	15.301	15.301	1	40.583	19.616	6.994e-05
TMS session	6.353	3.177	2	82.741	4.072	0.021
Time	8.134	8.134	1	100.365	10.428	0.002
Biotype * TMS session	11.678	5.839	2	71.488	7.485	0.001
**Follow-up contrasts**						
Cognitive biotype +						
Contrast	Estimate	SE	DF	t	p	95% CI
Early Treatment - Baseline	1.324	0.277	73.7	4.784	0.0001	[0.773; 1.876]
Post-Treatment - Baseline	1.623	0.485	99.1	3.347	0.001	[0.661; 2.585]
Post-Treatment - Early Treatment	0.299	0.413	93.5	0.722	0.472	[-0.523; 1.120]
Cognitive biotype -						
Contrast	Estimate	SE	DF	t	p	95% CI
Early Treatment - Baseline	-0.188	0.324	68.7	-0.581	0.563	[-0.834; 0.458]
Post-Treatment - Baseline	0.501	0.504	95	0.995	0.322	[-0.499; 1.501]
Post-Treatment - Early Treatment	0.689	0.453	88.5	1.521	0.132	[-0.211; 1.590]

Supplementary Table 2: Results of the mixed linear model predicting change in Go-NoGo performance. The cognitive biotype was defined by participants who have hypo-connected Left dlPFC-dACC NoGo > Go connectivity, less than 0 (N=26). We label these participants as "cognitive biotype +”. By contrast, those not in the cognitive biotype were defined by participants who have relatively intact connectivity, greater or equal to 0 (N=17). We label these participants as "cognitive biotype -”. Time refers to days from the baseline session on the day of the dependent variable assessment. All statistical tests were two-sided and not adjusted for multiple comparisons. *Abbreviations*: CI=confidence interval, dACC=dorsal anterior cingulate cortex, DF=degrees of freedom, dlPFC=dorsolateral prefrontal cortex, TMS=transcranial magnetic stimulation.

**Model effects**						
Effect	Sum Squares	Mean Squares	DF	Errors DF	F	p
Cognitive biotype – and +	4.546	4.546	1	31.550	7.238	0.011
TMS session	5.179	2.589	2	58.122	4.122	0.021
Time	5.235	5.235	1	73.273	8.334	0.005
Biotype * TMS session	5.248	2.624	2	51.949	4.178	0.021
**Follow-up contrasts**						
Cognitive biotype +						
Contrast	Estimate	SE	DF	t	p	95% CI
Early Treatment - Baseline	1.068	0.276	51.6	3.862	0.0003	[0.513; 1.62]
Post-Treatment - Baseline	2.054	0.646	68.6	3.177	0.002	[0.764; 3.34]
Post-Treatment - Early Treatment	0.986	0.568	66.7	1.737	0.087	[-0.147; 2.12]
Cognitive biotype -						
Contrast	Estimate	SE	DF	t	p	95% CI
Early Treatment - Baseline	-0.072	0.337	50.6	-0.213	0.832	[-0.749; 0.60]
Post-Treatment - Baseline	1.211	0.632	66.7	1.916	0.060	[-0.051; 2.47]
Post-Treatment - Early Treatment	1.283	0.527	63.7	2.435	0.018	[0.230; 2.33]

Supplementary Table 3: Results of the mixed linear model predicting change in depression severity measured by QIDS total.

The cognitive biotype was defined by participants who have hypo-connected Left dlPFC-dACC NoGo > Go connectivity, less than 0 (N=26). We label these participants as "cognitive biotype +". By contrast, those not in the cognitive biotype were defined by participants who have relatively intact connectivity, greater or equal to 0 (N=17). We label these participants as "cognitive biotype -". Time refers to days from the baseline session on the day of the dependent variable assessment. All statistical tests were two-sided and not adjusted for multiple comparisons. *Abbreviations*: CI=confidence interval, dACC=dorsal anterior cingulate cortex, DF=degrees of freedom, dlPFC=dorsolateral prefrontal cortex, QIDS=Quick Depression Inventory Scale, TMS=transcranial magnetic stimulation.

**Model effects**						
Effect	Sum Squares	Mean Squares	DF	Errors DF	F	p
Cognitive biotype – and +	0.705	0.705	1	39.512	0.093	0.762
TMS session	66.296	33.148	2	84.361	4.366	0.016
Time	14.726	14.726	1	104.997	1.940	0.167
Cognitive biotype * TMS session	0.566	0.283	2	71.958	0.037	0.963
**Follow-up contrasts**						
Contrast	Estimate	SE	DF	t	p	95% CI
Early Treatment - Baseline	-1.880	0.683	75.000	-2.758	0.007	[-3.24; -0.523]
Post-Treatment - Baseline	-3.370	1.441	103.000	-2.337	0.021	[-6.23; -0.510]
Post-Treatment - Early Treatment	-1.480	1.209	101.000	-1.227	0.223	[-3.88; 0.916]

Supplementary Table 4: Responders and remitters post-treatment.
The cognitive biotype was defined by participants who have hypo-connected Left dlPFC-dACC NoGo > Go connectivity, less than 0 (N=26). We label these participants as "cognitive biotype +". By contrast, those not in the cognitive biotype were defined by participants who have relatively intact connectivity, greater or equal to 0 (N=17). We label these participants as "cognitive biotype -". Response was defined as a decrease of QIDS total of >50%, remission was considered as a post-treatment QIDS≤5. All statistical tests were two-sided and not adjusted for multiple comparisons.
Abbreviations: CI=confidence interval, dACC=dorsal anterior cingulate cortex, dlPFC=dorsolateral prefrontal cortex, QIDS=Quick Depression Inventory Scale.

	Biotype	
Response	Cognitive biotype +	Cognitive biotype -
Non-responders	13 (69%)	11 (75%)
Responders	6 (31%)	2 (15%)
Remission		
Non-remitters	16 (84%)	12 (92%)
Remitters	3 (16%)	1 (8%)

Supplementary Table 5: Results of the mixed linear model predicting change in connectivity between the left dlPFC and dACC accounting for left dlPFC activation.

The cognitive biotype was defined by participants who have hypo-connected Left dlPFC-dACC NoGo > Go connectivity, less than 0 (N=26). We label these participants as "cognitive biotype +”. By contrast, those not in the cognitive biotype were defined by participants who have relatively intact connectivity, greater or equal to 0 (N=17). We label these participants as "cognitive biotype -”. Time refers to days from the baseline session on the day of the dependent variable assessment. All statistical tests were two-sided and not adjusted for multiple comparisons. *Abbreviations*: CI=confidence interval, dACC=dorsal anterior cingulate cortex, DF=degrees of freedom, dlPFC=dorsolateral prefrontal cortex, TMS=transcranial magnetic stimulation.

**Model effects**						
Effect	Sum Squares	Mean Squares	DF	Errors DF	F	p
Cognitive biotype – and +	15.286	15.286	1	40.396	19.442	7.489e-05
TMS session	5.637	2.818	2	82.787	3.585	0.032
Left dlPFC NoGo > Go activation	0.121	0.121	1	97.186	0.154	0.696
Time	7.605	7.605	1	98.778	9.673	0.002
Cognitive biotype * TMS session	11.630	5.815	2	71.714	7.397	0.001

**Follow-up contrasts**						
Cognitive biotype +						
Contrast	Estimate	SE	DF	t	p	95% CI
Early Treatment - Baseline	1.301	0.284	76.1	4.574	0.001e-21	[0.735; 1.868]
Post-Treatment - Baseline	1.597	0.492	98.7	3.248	0.002	[0.621; 2.573]
Post-Treatment - Early Treatment	0.296	0.415	92.6	0.713	0.478	[-0.529; 1.121]
Cognitive biotype -						
Contrast	Estimate	SE	DF	t	p	95% CI
Early Treatment - Baseline	-0.201	0.327	68.6	-0.615	0.540	[-0.853; 0.451]
Post-Treatment - Baseline	0.457	0.519	95.8	0.880	0.381	[-0.573; 1.487]
Post-Treatment - Early Treatment	0.658	0.462	89.3	1.423	0.158	[-0.261; 1.577]

Supplementary Table 6: Results of the mixed linear model predicting change in Go-NoGo performance accounting for left dlPFC activation.
The cognitive biotype was defined by participants who have hypo-connected Left dlPFC-dACC NoGo > Go connectivity, less than 0 (N=26). We label these participants as "cognitive biotype +". By contrast, those not in the cognitive biotype were defined by participants who have relatively intact connectivity, greater or equal to 0 (N=17). We label these participants as "cognitive biotype -". Time refers to days from the baseline session on the day of the dependent variable assessment. All statistical tests were two-sided and not adjusted for multiple comparisons. *Abbreviations*: CI=confidence interval, dACC=dorsal anterior cingulate cortex, DF=degrees of freedom, dlPFC=dorsolateral prefrontal cortex, TMS=transcranial magnetic stimulation.

**Model effects**						
Effect	Sum Squares	Mean Squares	DF	Errors DF	F	p
Cognitive biotype – and +	3.112	3.112	1	30.752	6.237	0.018
TMS session	1.644	0.822	2	53.834	1.647	0.202
Left dlPFC NoGo > Go activation	1.017	1.017	1	66.977	2.039	0.158
Time	1.220	1.220	1	68.989	2.445	0.122
Biotype * TMS session	3.080	1.540	2	47.859	3.087	0.055
**Follow-up contrasts**						
Cognitive biotype +						
Contrast	Estimate	SE	DF	t	p	95% CI
Early Treatment - Baseline	0.766	0.265	51.5	2.892	0.006	[0.235; 1.298]
Post-Treatment - Baseline	1.327	0.695	67.3	1.910	0.060	[-0.060; 2.714]
Post-Treatment - Early Treatment	0.561	0.611	64.8	0.918	0.362	[-0.659; 1.781]
Cognitive biotype -						
Contrast	Estimate	SE	DF	t	p	95% CI
Early Treatment - Baseline	-0.150	0.310	46.8	-0.486	0.629	[-0.773; 0.472]
Post-Treatment - Baseline	0.773	0.620	66.6	1.247	0.217	[-0.464; 2.011]
Post-Treatment - Early Treatment	0.924	0.520	63.6	1.778	0.080	[-0.114; 1.962]

Supplementary Table 7: Results of the mixed linear model predicting change in depression severity measured by QIDS total accounting for left dlPFC activation.

The cognitive biotype was defined by participants who have hypo-connected Left dlPFC-dACC NoGo > Go connectivity, less than 0 (N=26). We label these participants as "cognitive biotype +". By contrast, those not in the cognitive biotype were defined by participants who have relatively intact connectivity, greater or equal to 0 (N=17). We label these participants as "cognitive biotype -". Time refers to days from the baseline session on the day of the dependent variable assessment. All statistical tests were two-sided and not adjusted for multiple comparisons. *Abbreviations*: CI=confidence interval, dACC=dorsal anterior cingulate cortex, DF=degrees of freedom, dlPFC=dorsolateral prefrontal cortex, QIDS=Quick Depression Inventory Scale, TMS=transcranial magnetic stimulation.

**Model effects**						
Effect	Sum Squares	Mean Squares	DF	Errors DF	F	p
Cognitive biotype – and +	0.064	0.064	1	41.418	0.009	0.924
TMS session	46.323	23.161	2	80.217	3.371	0.039
Left dlPFC NoGo > Go activation	2.523	2.523	1	94.544	0.367	0.546
Time	20.640	20.640	1	95.942	3.004	0.086
Biotype * TMS session	0.302	0.151	2	69.923	0.022	0.978
**Follow-up contrasts**						
Contrast	Estimate	SE	DF	t	p	95% CI
Early Treatment - Baseline	-1.69	0.688	72.2	-2.458	0.016	[-3.06; -0.320]
Post-Treatment - Baseline	-2.99	1.495	95.1	-1.996	0.049	[-5.95; -0.017]
Post-Treatment - Early Treatment	-1.29	1.245	92.9	-1.039	0.301	[-3.77; 1.179]

Supplementary Table 8: Results of the mixed linear model predicting change in connectivity between the left dlPFC and dACC accounting for motion.
The cognitive biotype was defined by participants who have hypo-connected Left dlPFC-dACC NoGo > Go connectivity, less than 0 (N=26). We label these participants as "cognitive biotype +". By contrast, those not in the cognitive biotype were defined by participants who have relatively intact connectivity, greater or equal to 0 (N=17). We label these participants as "cognitive biotype -". Time refers to days from the baseline session on the day of the dependent variable assessment. Motion was calculated as the number of volumes showing framewise displacement ≥ 0.3. All statistical tests were two-sided and not adjusted for multiple comparisons.
Abbreviations: CI=confidence interval, dACC=dorsal anterior cingulate cortex, DF=degrees of freedom, dlPFC=dorsolateral prefrontal cortex, TMS=transcranial magnetic stimulation.

**Model effects**						
Effect	Sum Squares	Mean Squares	DF	Errors DF	F	p
Cognitive biotype – and +	15.718	15.718	1	39.334	20.694	5.0627e-05
TMS session	6.750	3.375	2	81.261	4.443	0.015
Motion	1.941	1.941	1	61.091	2.555	0.115
Time	8.838	8.838	1	99.974	11.635	0.001
Biotype * TMS session	11.568	5.784	2	70.586	7.615	0.001
**Follow-up contrasts**						
Cognitive biotype +						
Contrast	Estimate	SE	DF	t	p	95% CI
Early Treatment - Baseline	1.322	0.273	73.1	4.835	0.1e-23	[0.777; 1.867]
Post-Treatment - Baseline	1.696	0.483	96.9	3.514	0.001	[0.738; 2.654]
Post-Treatment - Early Treatment	0.374	0.412	90.7	0.907	0.367	[-0.445; 1.193]
Cognitive biotype -						
Contrast	Estimate	SE	DF	t	p	95% CI
Early Treatment - Baseline	-0.192	0.320	68.2	-0.602	0.549	[-0.830; 0.445]
Post-Treatment - Baseline	0.606	0.503	92.2	1.203	0.232	[-0.394; 1.605]
Post-Treatment - Early Treatment	0.798	0.454	86.0	1.758	0.082	[-0.105; 1.701]

Supplementary Table 9: Results the mixed linear model predicting change in Go-NoGo performance accounting for motion.

The cognitive biotype was defined by participants who have hypo-connected Left dlPFC-dACC NoGo > Go connectivity, less than 0 (N=26). We label these participants as "cognitive biotype +". By contrast, those not in the cognitive biotype were defined by participants who have relatively intact connectivity, greater or equal to 0 (N=17). We label these participants as "cognitive biotype -". Time refers to days from the baseline session on the day of the dependent variable assessment. Motion was calculated as the number of volumes showing framewise displacement ≥ 0.3. All statistical tests were two-sided and not adjusted for multiple comparisons.

Abbreviations: CI=confidence interval, dACC=dorsal anterior cingulate cortex, DF=degrees of freedom, dlPFC=dorsolateral prefrontal cortex, TMS=transcranial magnetic stimulation.

**Model effects**						
Effect	Sum Squares	Mean Squares	DF	Errors DF	F	p
Cognitive biotype – and +	1.995	1.995	1	25.217	3.791	0.063
TMS session	1.392	0.696	2	53.074	1.322	0.275
Motion	2.882	2.881	1	33.946	5.475	0.025
Time	0.785	0.785	1	68.985	1.492	0.226
Biotype * TMS session	3.388	1.694	2	48.115	3.218	0.049
**Follow-up contrasts**						
Cognitive biotype +						
Contrast	Estimate	SE	DF	t	p	95% CI
Early Treatment - Baseline	0.803	0.263	49.4	3.057	0.004	[0.275; 1.331]
Post-Treatment - Baseline	1.092	0.701	68.3	1.558	0.124	[-0.306; 2.490]
Post-Treatment - Early Treatment	0.289	0.622	67.4	0.464	0.644	[-0.953; 1.530]
Cognitive biotype -						
Contrast	Estimate	SE	DF	t	p	95% CI
Early Treatment - Baseline	-0.159	0.315	47.3	-0.505	0.616	[-0.792; 0.474]
Post-Treatment - Baseline	0.583	0.625	65.8	0.932	0.355	[-0.665; 1.831]
Post-Treatment - Early Treatment	0.741	0.530	62.9	1.399	0.167	[-0.317; 1.800]

Supplementary Table 10: Results of the mixed linear model predicting change in depression severity measured by QIDS total accounting for motion.

The cognitive biotype was defined by participants who have hypo-connected Left dlPFC-dACC NoGo > Go connectivity, less than 0 (N=26). We label these participants as "cognitive biotype +". By contrast, those not in the cognitive biotype were defined by participants who have relatively intact connectivity, greater or equal to 0 (N=17). We label these participants as "cognitive biotype -". Time refers to days from the baseline session on the day of the dependent variable assessment. Motion was calculated as the number of volumes showing framewise displacement ≥ 0.3. All statistical tests were two-sided and not adjusted for multiple comparisons.

Abbreviations: CI=confidence interval, dACC=dorsal anterior cingulate cortex, DF=degrees of freedom, dlPFC=dorsolateral prefrontal cortex, QIDS=Quick Depression Inventory Scale, TMS=transcranial magnetic stimulation.

**Model effects**						
Effect	Sum Squares	Mean Squares	DF	Errors DF	F	p
Cognitive biotype – and +	0.211	0.211	1	41.334	0.031	0.862
TMS session	41.774	20.887	2	78.746	3.054	0.053
Motion	3.677	3.677	1	65.783	0.538	0.466
Time	27.242	27.242	1	94.813	3.984	0.049
Biotype * TMS session	0.683	0.342	2	69.927	0.050	0.951
**Follow-up contrasts**						
Contrast	Estimate	SE	DF	t	p	95% CI
Early Treatment - Baseline	-1.59	0.668	68.5	-2.379	0.020	[-2.92; -0.257]
Post-Treatment - Baseline	-2.60	1.470	91.4	-1.767	0.081	[-5.52; 0.322]
Post-Treatment - Early Treatment	-1.01	1.249	88.9	-0.807	0.422	[-3.49; 1.475]

Supplementary Table 11: Healthy control sample characteristics.
Healthy controls (N=144) were used as a reference group to express imaging data in terms of standard deviations from a healthy norm.
Abbreviations: SD=standard deviation.

N	144
Age (mean, SD)	32.16 (12.45)
Race, n (%)	
White	100 (69.4%)
Black	1 (0.7%)
Asian	32 (22.2%)
Multiracial	4 (2.8%)
Other	7 (4.9%)
Ethnicity, n (%)	
Hispanic	16 (11.1%)
Not Hispanic	59 (50.0%)
Information not available	69 (47.9%)
Gender, n (%)	
Male	73 (50.7%)
Female	71 (49.3%)
Highest Education, n (%)	
High School	21 (14.6%)
Some College	11 (7.6%)
College	45 (31.3%)
More than College	38 (26.4%)
Information not available	29 (20.1%)
Sites, n (%)	
Palo Alto	76 (52.8%)
Sydney	68 (47.2%)
